# Comparative differential proteomic profiles of nonfailing and failing hearts after in vivo thoracic aortic constriction in mice overexpressing FKBP12.6

**DOI:** 10.1002/phy2.39

**Published:** 2013-07-29

**Authors:** Miresta Prévilon, Morgane Le Gall, Philippe Chafey, Christian Federeci, Mylène Pezet, Guilhem Clary, Cédric Broussard, Guillonneau François, Jean-Jacques Mercadier, Patricia Rouet-Benzineb

**Affiliations:** 1 UMR698, INSERMParis, France; 2 U1016, Institut Cochin, Plate-forme protéomique, INSERMParis, France; 3 CRI Inserm/UJF U823, INSERMLa Tronche, France; 4 U1016, Institut Cochin, Plate-forme protéomique, INSERMParis, France; 5 U1016, Institut Cochin, Plateforme protéomique, Univ Paris DescartesParis, France; 6 CEFI-Institut Claude Bernard-IFR2, INSERM U698AP-HP, Hosp Bichat, Paris, France

**Keywords:** 2D-DIGE, cardiac hypertrophy, FKBP12.6, gender, heart failure, pressure overload, Proteomics, transgenic mice

## Abstract

Chronic pressure overload (PO) induces pathological left ventricular hypertrophy (LVH) leading to congestive heart failure (HF). Overexpression of FKBP12.6 (FK506-binding protein [K]) in mice should prevent Ca2+-leak during diastole and may improve overall cardiac function. In order to decipher molecular mechanisms involved in thoracic aortic constriction (TAC)-induced cardiac remodeling and the influence of gender and genotype, we performed a proteomic analysis using two-dimensional differential in-gel electrophoresis (2D-DIGE), mass spectrometry, and bioinformatics techniques to identify alterations in characteristic biological networks. Wild-type (W) and K mice of both genders underwent TAC. Thirty days post-TAC, the altered cardiac remodeling was accompanied with systolic and diastolic dysfunction in all experimental groups. A gender difference in inflammatory protein expression (fibrinogen, α-1-antitrypsin isoforms) and in calreticulin occurred (males > females). Detoxification enzymes and cytoskeletal proteins were noticeably increased in K mice. Both non- and congestive failing mouse heart exhibited down- and upregulation of proteins related to mitochondrial function and purine metabolism, respectively. HF was characterized by a decrease in enzymes related to iron homeostasis, and altered mitochondrial protein expression related to fatty acid metabolism, glycolysis, and redox balance. Moreover, two distinct differential protein profiles characterized TAC-induced pathological LVH and congestive HF in all TAC mice. FKBP12.6 overexpression did not influence TAC-induced deleterious effects. Huntingtin was revealed as a potential mediator for HF. A broad dysregulation of signaling proteins associated with congestive HF suggested that different sets of proteins could be selected as useful biomarkers for HF progression and might predict outcome in PO-induced pathological LVH.

## Introduction

Aortic stenosis causes chronic pressure overload (PO) of the left ventricle (LV) that induces myocardial remodeling. In response to the increase in hemodynamic load and neurohormonal stress, the heart initially undergoes an adaptive compensatory left ventricular hypertrophy (LVH) that may progress to maladaptive hypertrophy which, in turn, may eventually lead, with time, to heart failure (HF). Impairment in Ca^2+^ homeostasis, such as the downregulation of Ca^2+^-modulating proteins (e.g., sarcoplasmic reticulum [SR] Ca^2+^-ATPase 2a: SERCA2a), is a common observation in HF. Among the multiple partners mediating the cardiac hypertrophy program and orchestrating Ca^2+^ homeostasis in the cardiomyocyte, FK506-binding proteins (FKBPs), ubiquitously expressed immunophilins (Schreiber 1991) are involved in the regulation of gene transcription, protein translation, and cell trafficking (Harrar et al. 2001). In particular, the two smallest members of the FKBP family, FKBP12 (calstabin1) and FKBP12.6 (calstabin2), both expressed in cardiomyocytes (Lam et al.^1995^), play a crucial role in the regulation of intracytoplasmic SR Ca^2+^ release (Marks 1996; Prestle et al. 2001). The affinity of FKBP12.6 for the Ca^2+^ release channel (ryanodine receptor [RyR2]) is higher than that of FKBP12 (Jeyakumar et al. 2001) and stabilizes RyR2 in a closed state during diastole, thereby reducing diastolic Ca^2+^ leak from the SR (Prestle et al. 2001). Disruption of the FKBP12.6 gene in mice results in cardiac hypertrophy in male, but not in female mice (Xin et al. 2002). FKPB12.6-null mice display no structural or functional abnormalities at rest (Wehrens et al. 2003). Indeed, the ablation of FKBP12.6 did not render mice susceptible to stress-induced ventricular arrhythmias (Xiao et al. 2007).

In our transgenic mouse model with specific cardiac overexpression of FKBP12.6 (Gellen et al. 2008), mice of both genders are healthy with no apparent morphologic abnormalities. In male mice we showed that FKBP12.6 overexpression in cardiomyocytes prevents triggered ventricular tachycardia in normal heart in stress conditions, this antiarrhythmic effect resulting likely from increased FKBP12.6 binding to RyR2 (Gellen et al. 2008). More recently, we showed that the cardiac overexpression of FKBP12.6 also protects against catecholamine-promoted burst pacing-induced ventricular tachycardia in the remodeled heart 2 months after thoracic aorta constriction (TAC) (Vinet et al. 2012). This is due at least in part to a decreased sensitivity of cardiomyocytes to catecholamines associated with decreased activation of the Ca2+/Calmodulin-dependent kinase II (decreased phospho-Ser ^2814^ RyR2) and calcineurin-nuclear factor of activated T cells signaling pathways (Prévilon et al. 2011).

Although much is known about the pathways that promote hypertrophic responses, biological functions can rarely be attributed to individual molecules, but rather arise through complex interactions between numerous cell components. We have shown that FKBP12.6 transgenic mice are protected against TAC-induced mortality, and that female mice develop hypertrophy and HF earlier than males (Prévilon et al. 2011). The latter was associated with temporal changes in Ca^2+^-dependent and independent cell processes but FKBP12.6 overexpression did not significantly affect the progression of HF following TAC. However, pathological hypertrophy eventually leads to HF via mechanisms that are not yet completely understood and the gender difference in cardiac dysfunction caused by TAC has not yet been clarified. In an effort to elucidate such mechanisms, the aim of this study was to build upon our previous findings to determine whether FKBP12.6 overexpression influences the cardiac remodeling proteome. Using the validated TAC model in mice to induce progressive hypertrophy leading with time to HF, we applied proteomic technology to investigate how FKBP12.6 overexpression could imprint the proteome signature in PO-induced remodeling of mice without (C) or mice with congestive heart failure (H).

## Material and Methods

### Experimental animals

The transgenic mice overexpressing FKBP12.6 on a B6D2/F1 background were produced in our laboratory and have been described in detail previously (Gellen et al. 2008). Male and female wild type (W) and mice overexpressing FKBP12.6 (K) were used. Mice were housed in a specific pathogen-free facility and handled in accordance with European Union Directives (86/609/EEC) on care and use of laboratory animals. The review and approval of the study was obtained by the local Animal Ethics Committee (No. B 7518 03).

### Transverse aortic constriction

Adult female and male mice (5–6 weeks old) of different genotypes (W or K), weighing (18–22 g), underwent TAC using a 27-gauge needle as previously described (Prévilon et al. 2010). Animals were killed 30 days later. Hearts were quickly excised; then chambers (atria, right, and LVs) were dissected, weighed, and immediately frozen in liquid nitrogen and stored at −80°C until use.

**Figure 1:**
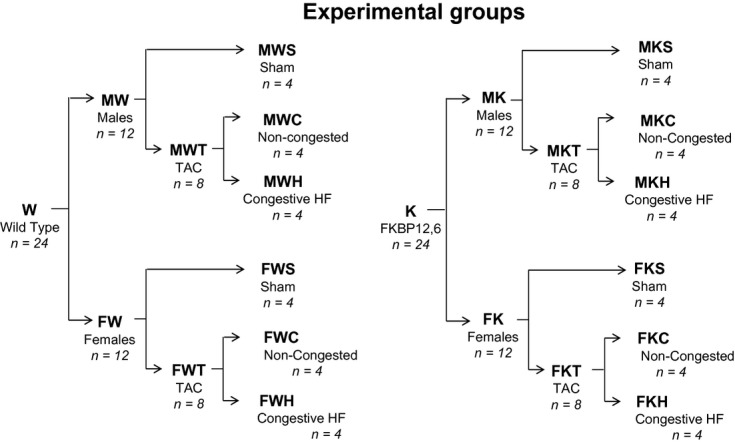
Experimental animal groups. The cardiac left ventricle from mice of both genders (*n* = 24) and genotypes (*n* = 24) were studied at 30 days postsurgery. Symbols used for genotypes are W (wild type) and K (mice overexpressing FKBP12.6), and for genders F (female) and M (male), sham-operated mice without banding (S) and mice having undergone thoracic aortic constriction (T for TAC). Due to the presence of pulmonary edema, two distinct TAC groups were defined as follows: without [noncongested mice, (C)] or with lung congestion [mice with congestive heart failure, (H)].

Experimental groups for two-dimensional differential in-gel electrophoresis (2D-DIGE) consisted of 12 groups (with four mice per group): female (F) and male (M) mice of the two genotypes were submitted either to TAC (T) or to sham operation (S). TAC groups were further split into two groups according to the presence or absence of lung edema, mice without pulmonary edema (noncongested, C) and mice with congestive heart failure (H). The abbreviations of designated groups are reported in Figure 1.

### Echocardiography

Transthoracic echocardiography was performed with a Toshiba Powervision 6000 (SSA 370A; Toshiba, Tokyo, Japan) device equipped with an 8- to 14-MHz linear and a 6- to 10-MHz sectoriel probe as previously described (Prévilon et al. 2011).

### Two-dimensional differential in-gel electrophoresis

To ensure reliability, all samples from the TAC group (C and H) and its respective sham-operated group (S) were processed simultaneously.

#### Tissue sample preparation

The frozen cardiac LVs from each mouse experimental group (*n* = 4 per group) were individually pulverized under liquid nitrogen to yield a fine powder using a pestle and mortar. The tissue powder was solubilized in lysis buffer (8 mol/L urea, 2 mol/L thiourea, 4% CHAPS, 60 mmol/L dithiothreitol [DTT]). Then the protein extracts were clarified by ultra-centrifugation at 100,000*g* for 1 h at 4°C. The supernatants were then treated with the 2D Clean-Up kit (GE Healthcare, Buc, France) according to the manufacturer's instructions. The resulting dry pellets were resuspended in lysis buffer without DTT and adjusted to pH 8.5 with 1 mol/L Tris-base. Protein concentrations of the samples were determined by the Bradford method, and were in the range of 9–12 μg/μL.

#### Two-dimensional differential in-gel electrophoresis

The LV Samples (50 μg) were labeled with CyDyes™ Fluor minimal dyes (GE Healthcare) Cy3 or Cy5 according to the manufacturer's instructions. The internal standard was prepared by combining equal quantities of all samples used for this study and labeled with Cy2. Fifty micrograms of labeled samples (Cy3 or Cy5) and internal standard (Cy2) were mixed as indicated in Table 1 and each mixed Cy-dye labeled protein extract (150 μg) was added to a rehydration buffer (8 mol/L urea, 2 mol/L thiourea, 2% (w/v) CHAPS, 10 mmol/L DTT, 1,2% (v/v) pH 4–7 IPG buffer (GE Healthcare) and trace of bromophenol blue). Immobiline™ Drystrips (pH 4–7, 18 cm, GE Healthcare) were rehydrated in the dark for 24 h at room temperature under low-viscosity paraffin oil. Isoelectric focusing was performed using an IPGphor system (GE Healthcare) for a total of 52 kVh. IPG strips were then incubated consecutively for 15 min each in equilibration buffer I and II (Buffer I: 50 mmol/L Tris-HCl, pH 8.8, 6 mol/L urea, 2% [w/v] sodium dodecyl sulfate [SDS], 30% [v/v] glycerol and 1% [w/v] DTT; Buffer II: Buffer I with 4.7% [w/v] iodoacetamide and no DTT). Equilibrated strips were placed onto homemade 12% SDS-polyacrylamide gel (SDS-PAGE) and overlaid with agarose solution (0.5% low-melting agarose with a trace of bromophenol blue in running buffer) and electrophoresis was performed in a Ettan-DALT II system (GE Healthcare) at 2.5 W/gel at 12°C until the bromophenol blue dye reached the bottom of the gel. Low fluorescent glass plates were used to minimize background fluorescence during scanning. The gels were scanned using a Typhoon 9400 Trio Variable Mode Imager (GE Healthcare). Gels were scanned using optimal excitation/emission wavelength for each DIGE fluor (Cy2 488/520 nm; Cy3 532/580 nm; Cy5 633/670 nm) and with a resolution set at 100 μm.

**Table 1:** Experimental design for 2D-DIGE analysis

Gel number	Cy3 labeling (50 μg protein)	Cy5 labeling (50 μg protein)	Cy2 labeling (50μg protein)	Number of detected spots	Percentage of matched spot referenced to gel 1
1	FWH1	FKS3	IS	1795	100%
2	FWH2	FKC3	IS	1634	77%
3	FWC1	FKH3	IS	1630	79%
4	FWC2	FKS4	IS	1546	76%
5	FWS1	FKC4	IS	1524	74%
6	FWS2	FKH4	IS	1543	77%
7	FKH1	FWH3	IS	1650	79%
8	FKH2	FWS3	IS	1505	75%
9	FKC1	FWC3	IS	1627	78%
10	FKC2	FWH4	IS	1541	75%
11	FKS1	FWC4	IS	1589	76%
12	FKS2	FWS4	IS	1519	73%
13	MWH1	MKS3	IS	1502	63%
14	MWH2	MKC3	IS	1741	75%
15	MWC1	MKH3	IS	1702	73%
16	MWC2	MKS4	IS	1572	67%
17	MWS1	MKC4	IS	1754	74%
18	MWS2	MKH4	IS	1733	72%
19	MKH1	MWH3	IS	1662	74%
20	MKH2	MWS3	IS	1562	70%
21	MKC1	MWC3	IS	1606	70%
22	MKC2	MWH4	IS	1490	68%
23	MKS1	MWC4	IS	1487	70%
24	MKS2	MWS4	IS	1672	73%

Fifty micrograms of the proteins extracted from each sample were labeled with Cy3 or Cy5 as indicated. Cy2-labeled Internal Standard (IS) was prepared by combining equal portions of the 48 left ventricular samples. Analytical gels of left ventricular proteins from MWS, male wild-type sham-operated mice (*n* = 4); MWC, male wild-type noncongested mice (*n* = 4); MWH, male wild-type mice with congestive heart failure (*n* = 4); FWS, female wild-type sham-operated mice (*n* = 4); FWC, female wild-type noncongested mice (*n* = 4); FWH, female wild-type mice with congestive heart failure (*n* = 4); MKS, male FKBP12.6 overexpressing sham-operated mice (*n* = 4); MKC, male FKBP12.6 overexpressing noncongested mice (*n* = 4); MKH, male FKBP12.6 overexpressing mice with congestive heart failure (*n* = 4); FKS, female FKBP12.6 overexpressing sham-operated mice (*n* = 4); FKC, female FKBP12.6 overexpressing noncongested mice (*n* = 4); FKH, female FKBP12.6 overexpressing mice with congestive heart failure (*n* = 4).

#### 2D-DIGE differential expression analysis

Image analysis, relative quantification and statistical evaluation and PCA (Principal Component Analysis) were carried out with DeCyder™ 2D software (GE Healthcare, version 7.0). The one-way analysis of variance (ANOVA) test followed by correction for false discovery rate (FDR) (*P* < 0.05) were used to determine protein spots significantly different between analyses. The fold change (FC) and Student's *T*-test *P*-values were calculated across several pairwise comparisons (Male [M] vs. Female [F], FKBP12. 6 [K] vs. Wild type [W], TAC [H, C] vs. Sham [S], congestive failing [H] vs. S, and noncongested mice [C] vs. S) and considered significant for *P*-values <0.05 and FC >1.2 or <−1.2, taking into account the power of DIGE method to detect a reliable difference in protein abundance down to 15% (Marouga et al. 2005; Viswanathan et al. 2006). FDR correction was applied as a multiple testing correction method to keep the overall error rate as low as possible (Benjamini and Hochberg 2000). Proteins of interest were identified by mass spectrometry.

### Protein identification by mass spectrometry

For mass spectrometry analysis, two semi-preparative 2D-gels were prepared as analytical gels. The IPG strips were rehydrated with 400 μg of equal amounts of male LV or female LV samples, respectively. After electrophoresis, 2D-gels were fixed in 30% (v/v) ethanol, 2% (v/v) phosphoric acid (two changes, 30 min each), and then stained for 72 h in 0.01% (w/v) Coomassie Brilliant Blue G-250, 12% (w/v) ammonium sulfate, 18% (v/v) ethanol, and 2% (v/v) phosphoric acid. Spots of interest were manually excised from Coomassie blue-stained semi-preparative gels. Destained and dehydrated gel spots were digested with trypsin (Promega) solution (12.5 ng/μL in 40 mmol/L NH_4_HCO_3_-10% acetonitrile) overnight at 40°C. Extracted Peptides were then desalted and concentrated using C_18_ ZipTips (Millipore, Molsheim, France). Samples were loaded onto a Ziptip by pipetting up and down ten times using a fresh 96-well plate. Samples (in ZipTips) were washed three times with 10 μL of 0.1% trifluoroacetic acid (TFA) and eluted with 4 μL of 50% acetonitrile/0.1% TFA and with 4 μL of 70% acetonitrile/0.1% TFA. Eluted peptides were pooled, dried, and a total of 1.5 μL of eluate was pipetted onto a clean Matrix-Assisted Laser Desorption/Ionisation plate covered with 1.5 μL of α-cyano-4-hydroxycinnamic acid MALDI matrix (LaserBioLabs, Sophia-Antipolis, France).

Mass spectra for each spot were acquired with a MALDI-TOF/TOF mass spectrometer (MALDI-TOF-TOF 4800, ABSciex, les Ulis, France), running version 3.5.28193 of 4000 series explorer software. After filtering tryptic-, keratin-, and matrix-contaminant peaks up to 15 parent ions were selected for subsequent MS/MS fragmentation according to mass range, signal intensity, signal to noise ratio, and absence of neighboring masses in the MS spectrum. Database searching was carried out using Mascot version 2.2 (MatrixScience, London, U.K.) via GPS explorer software (ABSciex) version 3.6 combining MS and MS/MS interrogations on *Mus musculus* from Swiss-Prot databank 57.13 containing 16271 sequences (January 2010) (http://www.expasy.org). The search parameters were as follows: carbamidomethylation as a variable modification for cysteine and oxidation as a variable modification for methionine residues. Up to 1 missed tryptic cleavage was permitted and mass accuracy tolerance of 30 ppm for precursors and 0.3 Da for fragments were used for all trypsic mass searches. Positive identification was based on a Mascot score above the significance level (i.e., <5%). The reported proteins were always those with the highest number of peptide matches. Under our identification criteria, no result was found to match multiple members of a protein family.

### Modeling with ingenuity pathway analysis

In order to gain insights into the biological pathways and networks that were significantly represented in our proteomic datasets (regulated proteins identified by 2D-DIGE and mass spectrometry) we used ingenuity pathway analysis (IPA; Ingenuity Systems, Redwood City, CA). http://www.ingenuity.com). IPA builds hypothetical networks from these focus proteins, and other non–2D DIGE-identified proteins. The networks are displayed graphically as nodes (individual proteins) and edges (the biologic relationships between the nodes). IPA computes a score for each network from the *P*-value that indicates the likelihood of the *focus proteins in a network being found together due to random chance*. We selected only networks scoring ≥2, with *P* < 0.01 of not being generated by chance. Biological functions were assigned to each network by use of annotations from the scientific literature and stored in the Ingenuity Pathways Knowledge Base (IPKB). The Fisher exact test was used to calculate the *P*-value determining the probability of each biological function/disease or pathway being assigned by chance. The build function of IPA allows for generating pathways that can complete the data analysis by showing interactions of identified proteins with a specific group of molecules.

### Western blotting

Fifty micrograms of each cardiac LV lysate were loaded onto a 4–20% gradient, 10 or 18% SDS-PAGE and transferred to nitrocellulose membranes. The membranes were blocked and immunoblotted with diluted primary antibodies to sarcolemmal membrane-associated protein (SLMAP) (1:1000, sc-100957, Santa Cruz, Biotechnology Inc., Santa Cruz, CA, USA), HSPB1 (anti-HSP27, 1:1000, sc1049 Santa Cruz, Biotechnology), CRYAB (1:2000, Thermo Fisher Scientific, Illkirch, France), NDRG2 (1:5000, ab72140, Abcam, Paris, France), ERp29 (1:2500, ab11420, Abcam), huntingtin (HTT) (1:1000, clone 1HU-4C8, MAB2166, Millipore), and finally incubated with horseradish peroxidase (HRP)-conjugated anti-mouse or anti-rabbit secondary antibodies, as appropriate. Visualization by chemiluminescence detection was carried out according to the manufacturer's instructions (ECL kit, GE Healthcare). Equal protein loading for LV lysates was assessed by stripping blot and reprobing with an anti-calsequestrin antibody (CSQ; 1: 2500, PA1-913, Affinity BioReagents, Golden, CO, USA). Quantitation of digitized images of immunoblots was done using ImageJ software (http://rsb.info.nih.gov/nih-image/about.html.). The intensity of immunoreactive bands was normalized to that of calsequestrin. Data are expressed as percentages of the respective control (means ± SEM).

### Coimmunoprecipitation

Immunoprecipitation (IP) buffer (50 mmol/L TRis-HCl pH7.4, 100 mmol/L NaCl, 15 mol/L EDTA, 1% Triton × 100) supplemented with a complete cocktail of inhibitor proteases (fast Sigma Aldrich Chimie, Saint-Quentin-Fallavier, France) was added to 200 μg of LV lysates to a final volume of 0.5 mL. The LV lysates were depleted in immunoglobulins by incubation with protein A- and protein G- Sepharose magnetic beads for 1 h at 4°C. Using Pierce Crosslink magnetic IP/Co-IP kit (Thermo Scientific, Illkirch, France), 5 μg of anti-HSP27 antibody (sc1049) were crosslinked onto protein A/G magnetic beads according to the manufacturer's instructions. The cleared lysates were incubated with HSP27 antibody-crosslinked beads on a rotator overnight at 4°C, then the beads were collected with a magnetic stand and nonbound sample was removed and saved for analysis. Following three washes of beads with IP-buffer, 100 μL of elution buffer was added and incubated under rotation for 5 min at room temperature. The beads were magnetically separated and the pH of elution buffer containing the IP-target antigen was adjusted by adding 1 mol/L Tris, pH 7.5. Laemmli loading buffer was added to the eluted protein. Immunoprecipitated protein samples were fractioned on 3–8% NuPAGE Tris-Acetate gel (Life technologies, Saint Aubin, France), then transferred to nitrocellulose membrane. After blocking, the membrane was incubated with primary antibodies to anti-HTT, 1:1000 or anti- IMMT, 1:5000. After 3 washes, the membranes were probed, respectively, with HRP-linked anti-mouse or -rabbit secondary antibodies. Co-IP protein was detected by chemiluminescence. Films were digitized and quantitated using ImageJ.

#### Statistical analysis

Results were presented as means ± SEM. To determine the effects of surgery, gender, or genotype and their interaction on expressed identified protein in LV, statistical differences were determined using ANOVAs followed by the FDR correction method (Benjamini and Hochberg, 1995) for multiple comparisons, *P* ≤ 0.05 indicates statistical significance.

## Results

### TAC induces pathological LV hypertrophy leading to congestive HF

**Figure 2:**
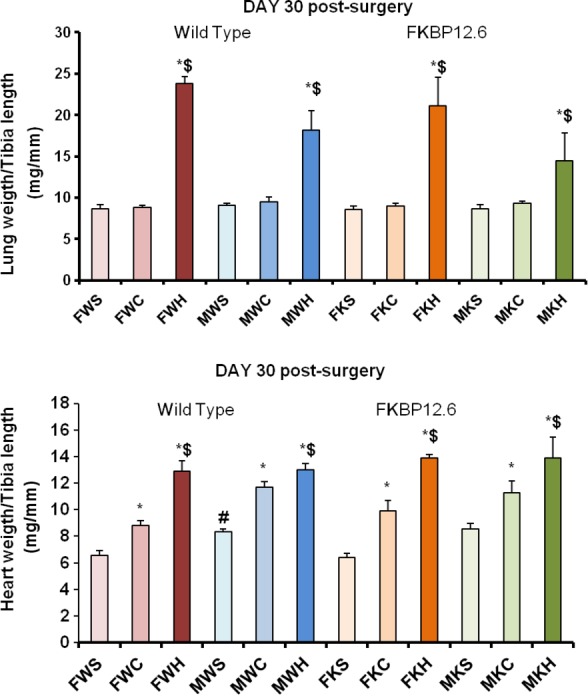
Characterization of the animal model. At 30 day postsurgery***,*** thoracic aortuc constriction (TAC) induced pathological left ventricular hypertrophy and congestive HF in wild-type mice (W) and in FKBP12.6 overexpressing mice (K) of both genders (M for male; F for female). Sham-operated mice (S) were also studied at 30 days. (A), histograms showing the ratio of lung weight to tibia length in groups where pulmonary edema index was used to classify TAC mice into noncongested mice (C) or mice with congestive heart failure (H). (B), hypertrophic responses to TAC, (heart weight to tibia length as hypertrophy index) for mice of both genotypes and genders. Data are means ± SEM; *P < 0.05 TAC compared with Sham; $*P* < 0.05, mice with lung congestion (H) compared with mice without lung congestion (C).

Gravimetric data are reported in Table 2. A significant gender difference in myocardial mass was observed in mice of both genotypes (W: wild type; K: FKBP12.6 overexpressing mice) without TAC, which was higher in male than in female mice (32 and 48%, respectively). Thirty days following surgery, heart weight to tibia length ratios were significantly increased in W and K mice with TAC compared with sham-operated mice (Table 2). Moreover, the presence of pulmonary edema (defined as lung weight to tibia length ratio in TAC mice > mean value in sham group + 3 SD) was used as the criterion to classify TAC mice. Accordingly, two groups were defined as noncongested mice (C) and as mice with congestive heart failure (H) (Fig. 2A). As expected, the hypertrophic response to TAC was more important in H than in C mice (Fig. 2B).

**Table 2:** Gravimetric data

		Female			Male		
			
Genotype		Sham	TAC		Sham	TAC	
		FWS	FWC	FWH	MWS	MWC	MWH
W	Mice (n)	4	4	4	4	4	4
	BW (g)	20 ± 1	22 ± 1	22 ± 1	31 ± 1	28 ± 0.2	28.4 ± 1.4
	LVW (mg)	79 ± 3	116 ± 6*	154 ± 9*	112 ± 4†	158 ± 4*	171 ± 2*
	HW/TL (mg/mm)	6.6 ± 0.3	8.8 ± 0.4*	13 ± 1*$	8.4 ± 0.2	12 ± 0.4*	13.0 ± 0.4*
	Lu W/TL (mg/mm)	8.6 ± 0.5	8.8 ± 0.3	24 ± 4*$	9.1 ± 0.3	9.5 ± 0.6	18.1 ± 2.4*$
K		FKS	FKC	FKH	MKS	MKC	MKH
	Mice (n)	4	4	4	4	4	4
	BW (g)	20 ± 1	23 ± 1	22 ± 1	28 ± 2	28 ± 1	25 ± 3
	LVW(mg)	73 ± 4	127 ± 10*	179 ± 2*	110 ± 10†	154 ±15	181 ± 18*
	HW/TL (mg/mm)	6.4 ± 0.4	9.9 ± 0.8*	14 ± 0.3*$	8.5 ± 0.4	11 ± 1*	13.9 ± 1.6*$
	Lu W/TL (mg/mm)	8.5 ± 0.5	9.9 ± 0.3*	21 ± 3*$	8.7 ± 0.4	9.3 ± 0.2*	14.4 ± 3.4*$

Data are mean ± SEM. W, wild-type mice; K, FKBP12.6 overexpressing mice; S, sham-operated; TAC, thoracic aortic constriction; F, female; M, male; C, noncongested mice; H, mice with congestive heart failure; BW, body weight; LVW; left ventricle weight; HW/TL, ratio heart weight to tibia length; Lu/TL, ratio lung weight to tibia length. *P < 0.05 TAC versus sham; $*P* < 0.05 C versus H mice; †*P* < 0.05 female versus male.

### Functional remodeling

Echocardiographic data are reported in Table 3. No difference was observed in stenotic jet indicating a similar degree of aortic constriction in all TAC mice (Table 3). Marked increases in interventricular septum (IVSTD) and posterior wall thickness (PWTD) at end diastole were found in female failing hearts of both genotypes. A significant genotype difference in the hypertrophic response to TAC was also observed. Male fKbp12.6 overexpressing TAC (MKT) mice exhibited a greater increase in IVSTD and PWTD than MWT mice. This increase was more pronounced in the MKH group. As expected, TAC was associated with alterations in LV function. In TAC mice, ejection fraction (EF) was reduced in both genders and genotypes, indicating systolic dysfunction. A genotype difference in the increase in LV end diastolic diameter (LVEDD) was also found in female mice, failing hearts being dilated in FK mice only. Early diastolic velocity of the mitral annulus (Ea) was decreased in all banded mice, whatever the gender or the genotype, indicating altered diastolic function. A gender difference was also observed in the TAC-induced decrease in aortic outflow (30 and 20% in male and female mice, respectively, *P* < 0.05). Thus, both genders of W and K mice developed LV hypertrophy following TAC accompanied with systolic and diastolic LV dysfunction.

**Table 3:** Echocardiographic data

		Female			Male		
			
Genotype		Sham	TAC		Sham	TAC	
W		FWS	FWC	FWH	MWS	MWC	MWH
	Mice (*n*)	4	5	3	5	4	4
	BW (g)	23 ± 1	21 ± 2	21 ± 1	25 ± 3	25 ± 2	25 ±1
	HR (bpm)	423 ± 38	420 ± 60	419 ± 38	456 ± 48	450 ± 50	435 ± 24
	IVSTD (mm)	0.57 ± 0.10	0.74 ± 0.07*	0.97 ± 0.07*$	0.61 ± 0.02	0.63 ± 0.18	0.68 ± 0.15
	PWTD (mm)	0.51 ± 0.06	0.80 ± 0.15*	1.12 ± 0.24*$	0.59 ± 0.10	0.77 ± 0.20*	0.76 ± 0.22
	LVmass (mg)	80.8 ± 9.4	140 ± 38*	211 ± 50*	99.9 ± 13.2	134.4 ± 20.7	164.6 ± 52.3
	LVEDD (mm)	4.3 ± 0.2	4.5 ± 0.4	4.5 ± 0.1	4.5 ± 0.4	4.8 ± 0.6	5.2 ± 0.1
	FS%	44 ± 7	32 ± 5*	28 ± 5*	39 ± 8	32 ± 2	22 ± 5*
	EF%	82 ± 6	68 ± 7*	63 ± 8*	77 ± 9	68 ± 3	52 ± 10*
	Spw (cm/sec)	3.1 ± 0.7	2.3 ± 0.1*	2.4 ± 0.5	3.1 ± 0.3	2.9 ± 0.4	2.3 ± 0.2*$
	Ea(cm/sec)	4.7 ± 0.5	3.3 ± 1.0*	3.2 ± 0.2*	4.6 ± 0.6	3.7 ± 0.6	2.9 ± 0.4*$
	E/Ea	0.2 ± 0.0	0.4 ± 0.1*	0.3 ± 0.0	0.2 ± 0.0	0.3 ± 0.0*	0.4 ± 0.0*$
	Aortic outflow (m/sec)	1.0 ± 0.2	0.8 ± 0.1	0.8 ± 0.1*	1.3 ± 0.2	1.0 ± 0.2 *	0.9 ± 0.0*$
	Stenotic jet velocity(m/sec)		4.5 ± 0.9	5.0 ± 0.2		4.4 ± 0.9	4.3 ± 1.1
	Velocity ratio		5.5 ± 1.0	6.5 ± 0.4		4.4 ± 1.4	4.9 ± 1.4
K		FKS	FKC	FKH	MKS	MKC	MKH
	Mice (*n*)	6	5	4	6	3	3
	BW (g)	24 ± 3	23 ± 2	22 ± 1	25 ± 2	24 ± 1	22 ± 2
	HR (bpm)	432 ± 18	417 ± 52	424 ± 33	450 ± 67	392 ± 49	359 ± 30
	IVSTD (mm)	0.58 ± 009	0.84 ± 0.08*	0.85 ± 0.14*	0.53 ± 0.07	0.71 ± 0.06	0.87 ± 0.04*
	PWTD (mm)	0.59 ± 0.14	0.95 ± 0.18*	1.03 ± 0.09*$	0.53 ± 0.08	140.6 ± 25.6*	0.95 ± 0.06*$
	LVmass (mg)	87.3 ± 27.0	165.7 ± 23.8*	205.9 ± 12.2*	88.9 ± 12.2	4.8 ± 0.3	188 ± 41*
	LVEDD (mm)	4.2 ± 0.3	4.5 ± 0.3	4.9 ± 02*$	4.6 ± 0.3	29 ± 3*	4.8 ± 0.7
	FS%	40 ± 5	37 ± 7	30 ± 4*	40 ± 6	65 ± 5*	26 ± 5*
	EF%	78 ± 6	74 ± 8	65 ± 6*	77 ± 6	2.2 ± 0.2*	60 ± 9*
	Spw (cm/sec)	2.8 ± 0.3	2.4 ± 8	2.4 ± 0.4	3.0 ± 0.6	4.0 ± 0.8	1.5 ± 0.3*
	Ea(cm/sec)	4.3 ± 0.4	3.2 ± 0.7*	3.0 ± 0.6*	4.0 ± 0.8	0.2 ± 0.1	2.1 ± 0.7*$
	E/Ea	0.2 ± 0.0	0.3 ± 0.1*	0.3 ± 0.0 *	0.3 ± 0.1	1.0 ± 0.0	0.4 ± 0.1*$
	Aortic outflow (m/sec)	1.0 ± 0.1	0.9 ± 0.1*	0.8 ± 0.1*	1.3 ± 0.2	3.8 ± 01	1.1 ± 0.0*$
	Stenotic jet velocity (m/sec)	–	4.4 ± 0.4	4.5 ± 0.4	–	3.7 ± 0.1	4.1 ± 1.3
	Velocity ratio	–	5.0 ± 0.7	5.8 ± 0.9	–		3.9 ± 1.2

W, wild-type mice; K, FKBP12.6 overexpressing mice; F, female; M, male; S, sham-operated; TAC, thoracic aortic constriction; C, noncongested mice; H mice with congestive heart failure; BW, body weight; TL, tibial length; HR, heart rate; IVSTD, interventricular septum thickness; PWTD, posterior wall thickness; LVEDD, LV end diastolic diameter; LV, left ventricle mass; EF, LV ejection fraction; Spw, maximum systolic velocity of posterior wall; E/Ea, peak velocity of early mitral inflow/early diastolic velocity of the mitral annulus; Velocity ratio, stenotic jet velocity/LV outflow velocity. Data are mean ± SEM. *P < 0.05, TAC versus sham; $*P* < 0.05, C versus H; †*P* < 0.05, female versus male.

### 2D-DIGE differential proteomic analysis

**Figure 3:**
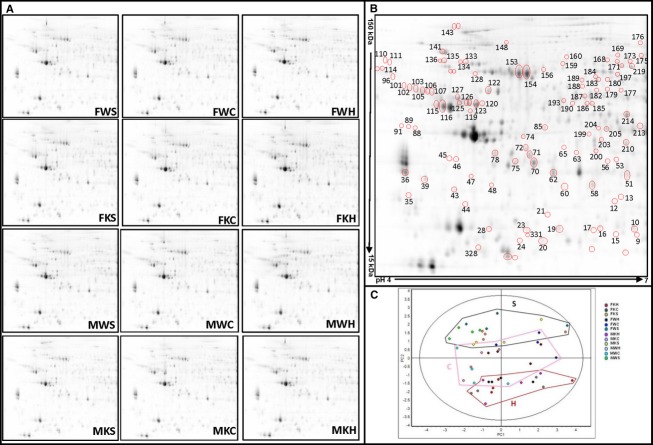
Mouse cardiac LV 2D-DIGE analysis. (A) Representative spot maps of each experimental group analyzed by 2D-DIGE. Protein extracts from cardiac left ventricles were prepared, labeled, and separated by 2D-DIGE, as described in Supplemental methods. (B) Differentially expressed protein spots, identified by DeCyder software, were identified by mass spectrometry (numbered/circled spots). Protein expression data were filtered by the following criteria: false discovery rate (FDR) and 1.2-fold difference in abundance. FDR correction was applied as a multiple testing correction method to keep the overall error rate as low as possible and *P*-value less than 0.05 in at least one of the following comparisons; MWH versus FWH; MWC versus FWC; MWS versus FWS; MKS versus FKS; MKH versus FKH; MKC versus FKC, FKH versus FWH; MKH versus MWH;FKC versus FWC; MKC versus MWC; FKS versus FWS; MKS versusMWS, FWC versus FWS; MWC versus MWS;FKC versusFKS; MKC versus MKS; FWH versus FWS; MWH versus MWS; FKH versus FKS; MKH versus MKS. (C) Principal component analysis (b>PCA) performed from the protein spots detected and matched. The score plot shows experimental maps.

Forty eight cardiac LV protein extracts, representing the 12 experimental groups (FWS [Female wild type sham-operated], FWC [Female wild type noncongested], FWH [Female wild type congestive heart failure], FKS [Female FKBP12.6 overexpressing Sham-operated], FKC [Female FKBP12.6 overexpressing noncongested], FKH [Female FKBP12.6 overexpressing congestive heart failure], MWS [Male wild type sham-operated], MWC [Male wild type noncongested], MWH [Male wild type congestive heart failure], MKS [Male FKBP12.6 overexpressing sham-operated], MKC [Male FKBP12.6 overexpressing noncongested], and MKH [Male FKBP12.6 overexpressing congestive heart failure]), were analyzed by 2D-DIGE technology. A representative set of 2D-DIGE gel images is shown in Figure 3A, and reveals the reproducibility of resolved protein maps, allowing the detection of 1610 ± 140 spots per gel, and 1312 ± 106 spots matched for the 24 2D-DIGE gels of the experiment.

In order to assess significant differential expression as a result of gender, genotype, and TAC, multiple group-to-group comparisons were performed (see Fig. 3B) using the DeCyder biological variation analysis (BVA) module. We identified a total of 96 spots differentially expressed by mass spectrometry (Table 4).

**Table 4:** MS/MS data

			Exp.		Theo.		Mass spectrometry			
					
Spot number	Protein name	Uniprot ID	pI	Mw	pI	Mw	Number of unique identified peptides in MSMS / in MS + MSMS	Total ion score	Total ion score	Sequence coverage (%)
4	ATP synthase subunit d, mitochondrial	Q9DCX2	5.5	18	5.5	19	8	612	116	60
6	Ferritin heavy chain	P09528	5.6	17	5.5	21	1	18	18	5
7	Ferritin heavy chain	P09528	5.6	18	5.5	21	2	40	21	10
8	Alpha-crystallin B chain	P23927	6.6	19	6.8	20	4	125	37	30
9	Glutathione S-transferase Mu 7	Q80W21	6.8	21	6.3	26	4	235	83	23
10	Glutathione S-transferase Mu 5	P48774	6.7	22	6.8	27	10	617	106	49
12	Glutathione S-transferase omega-1	O09131	6.5	27	6.9	27	2	40	24	10
17	Glutathione S-transferase Mu 5	P48774	6.3	22	6.8	27	7	284	76	35
18	Guanylate kinase	Q64520	6.3	19	6.1	22	2	44	22	9
20	Thioredoxin-dependent peroxide reductase, mitochondrial	P20108	5.8	20	7.2	28	3	119	70	14
21	Endoplasmic reticulum protein ERp29	P57759	5.9	25	5.9	29	1	16	16	3
23	Heat shock protein beta-1	P14602	5.7	22	6.1	23	6	311	110	32
28	Heat shock protein beta-1	P14602	5.3	22	6.1	23	5	308	111	28
30	14-3-3 protein beta/alpha	Q9CQV8	4.6	24	4.8	28	11	84	55	47
31	14-3-3 protein gamma	P61982	4.6	25	4.8	28	10	97	54	42
32	14-3-3 protein zeta/delta	P63101	4.6	24	4.7	28	10	301	85	49
35	Tropomyosin alpha-3 chain	P21107	4.6	29	4.7	33	1	22	22	3
39	Annexin A5	P48036	4.7	33	4.8	36	11	748	117	50
43	Ubiquinone biosynthesis protein COQ9, mitochondrial	Q8K1Z0	5.0	30	5.6	35	7	479	118	31
44	Chloride intracellular channel protein 1	Q9Z1Q5	5.1	27	5.1	27	6	287	64	34
45	Sarcolemmal membrane-associated protein	Q3URD3	5.0	38	5.2	67	2	60	39	2
46	Sarcolemmal membrane-associated protein	Q3URD3	5.0	38	5.2	67	7	286	66	9
47	Microtubule-associated protein RP/EB family member 2	Q8R001	5.2	33	5.2	37	2	50	29	6
48	F-actin-capping protein subunit beta	P47757	5.4	31	5.5	31	2	43	26	7
51	Electron transfer flavoprotein subunit alpha, mitochondrial	Q99LC5	6.7	33	8.6	35	8	600	109	37
53	PDZ and LIM domain protein 1	O70400	6.5	36	6.4	36	2	74	38	7
58	Delta(3,5)-Delta(2,4)-dienoyl-CoA isomerase, mitochondrial	O35459	6.3	31	7.6	36	7	404	92	25
60	Delta(3,5)-Delta(2,4)-dienoyl-CoA isomerase, mitochondrial	O35459	6.0	31	7.6	36	2	95	61	8
62	Malate dehydrogenase, cytoplasmic	P14152	5.9	34	6.2	36	6	377	91	21
63	Trans-1,2-dihydrobenzene-1,2-diol dehydrogenase	Q9DBB8	6.1	39	8.5	48	4	150	54	13
65	Leukocyte elastase inhibitor A	Q9D154	6.0	42	5.9	43	5	151	39	15
70	L-lactate dehydrogenase B chain	P16125	5.8	33	5.7	37	8	563	115	30
71	Isocitrate dehydrogenase	Q9D6R2	5.7	39	6.3	40	8	486	111	27
72	[Protein ADP-ribosylarginine] hydrolase-like protein 1	Q8BGK2	5.7	41	5.6	40	8	430	66	26
74	Serpin B6	Q60854	5.7	44	5.5	43	1	117	117	5
75	L-lactate dehydrogenase B chain	P16125	5.6	37	5.7	37	6	369	104	19
78	Isocitrate dehydrogenase	Q9D6R2	5.4	39	6.3	40	8	374	66	27
85	Adenosine kinase	P55264	5.8	47	5.8	40	8	454	81	25
89	Protein NDRG2	Q9QYG0	4.6	48	5.2	41	1	27	27	3
93	Calreticulin	P14211	4.3	70	4.3	48	4	159	72	12
96	Serine protease inhibitor A3K	P07759	4.4	68	5.1	47	3	138	55	12
101	Alpha-1-antitrypsin 1-4	Q00897	4.6	63	5.2	46	1	25	25	4
102	Alpha-1-antitrypsin 1-3	Q00896	4.6	63	5.2	46	4	152	77	16
103	Alpha-1-antitrypsin 1-1	P07758	4.7	62	5.4	46	6	268	74	25
104	Protein disulfide-isomerase	P09103	4.7	61	4.8	57	7	479	117	23
105	Alpha-1-antitrypsin 1-3	Q00896	4.7	62	5.2	46	7	380	99	29
106	Alpha-1-antitrypsin 1-1	P07758	4.8	61	5.4	46	7	402	105	26
107	Alpha-1-antitrypsin 1-2	P22599	4.8	61	5.3	46	7	410	114	22
110	Liver carboxylesterase N	P23953	4.4	75	5.1	61	1	27	27	2
115	ATP synthase subunit beta, mitochondrial	P56480	4.9	56	5.2	56	12	903	112	33
116	ATP synthase subunit beta, mitochondrial	P56480	4.9	55	5.2	56	12	1156	124	33
119	Dynactin subunit 2	Q99KJ8	5.2	54	5.1	44	5	189	52	15
120	Desmin	P31001	5.3	56	5.2	54	3	75	42	5
121	60 kDa heat shock protein, mitochondrial	P63038	5.3	56	5.9	61	8	424	91	21
122	60 kDa heat shock protein, mitochondrial	P63038	5.3	56	5.9	61	8	763	164	23
123	Desmin	P31001	5.2	56	5.2	54	13	1049	142	33
124	Desmin	P31001	5.2	56	5.2	54	13	848	108	32
125	Desmin	P31001	5.1	56	5.2	54	12	1017	128	30
126	Desmin	P31001	5.1	57	5.2	54	12	818	132	28
127	Vimentin	P20152	5.1	58	5.2	54	12	716	90	27
128	Actin, alpha skeletal muscle	P68134	5.2	68	5.2	42	3	146	69	10
130	NADH-ubiquinone oxidoreductase 75 kDa subunit, mitochondrial	Q91VD9	5.1	74	5.5	80	11	564	92	22
132	NADH-ubiquinone oxidoreductase 75 kDa subunit, mitochondrial	Q91VD9	5.1	74	5.5	80	4	77	22	8
133	Kelch repeat and BTB domain-containing protein 10	Q9ER30	5.0	70	5.0	68	6	206	41	11
134	Kelch repeat and BTB domain-containing protein 10	Q9ER30	5.0	70	5.0	68	3	57	24	4
135	Protein-glutamine gamma-glutamyltransferase 2	P21981	4.9	75	5.0	77	3	132	57	5
136	Protein-glutamine gamma-glutamyltransferase 2	P21981	4.9	75	5.0	77	3	103	43	4
143	Collagen alpha-1(VI) chain	Q04857	5.1	102	5.2	108	3	97	43	3
144	Collagen alpha-1(VI) chain	Q04857	5.1	100	5.2	108	5	170	50	6
148	Major vault protein	Q9EQK5	5.5	83	5.4	96	8	369	73	14
154	Serum albumin	P07724	5.7	68	5.8	69	14	1301	148	28
156	Serum albumin	P07724	5.8	69	5.8	69	2	46	23	4
168	Moesin	P26041	6.5	72	6.2	68	3	92	43	3
169	Serotransferrin	Q921I1	6.6	75	6.9	77	1	28	28	1
170	Moesin	P26041	6.6	72	6.2	68	5	182	66	7
173	Moesin	P26041	6.7	71	6.2	69	2	79	51	3
177	Propionyl-CoA carboxylase beta chain, mitochondrial	Q99MN9	6.6	59	7.2	58	2	98	60	4
179	Carboxylesterase 3	Q8VCT4	6.4	59	6.2	62	2	24	40	5
182	Fibrinogen beta chain	Q8K0E8	6.3	59	6.7	55	4	159	67	9
184	Dihydropyrimidinase-related protein 2	O08553	6.3	65	6.0	62	6	229	54	15
185	Aldehyde dehydrogenase, mitochondrial	P47738	6.3	55	7.5	57	4	161	59	8
186	Lipoamide acyltransferase component of branched-chain alpha-keto acid dehydrogenase complex, mitochondrial	P53395	6.3	54	8.9	53	4	141	40	10
187	T-complex protein 1 subunit beta	P80314	6.2	57	6.0	57	5	188	60	13
188	Adenylyl cyclase-associated protein 2	Q9CYT6	6.2	61	6.0	53	1	23	23	1
190	Aldehyde dehydrogenase, mitochondrial	P47738	6.1	54	5.7	57	4	41	14	8
191	Sarcalumenin	Q7TQ48	6.3	55	4.4	99	7	459	119	18
193	Selenium-binding protein 1	P17563	6.0	56	5.9	52	11	746	129	26
197	EH domain-containing protein 4	Q9EQP2	6.6	66	6.3	61	8	319	67	17
199	Septin-2	P42208	6.3	44	6.1	41	5	293	85	18
203	Short/branched chain specific acyl-CoA dehydrogenase, mitochondrial	Q9DBL1	6.4	42	8.0	47	4	160	66	13
204	Acyl-coenzyme A thioesterase 2, mitochondrial	Q9QYR9	6.4	46	6.9	50	4	145	51	13
205	Acyl-coenzyme A thioesterase 2, mitochondrial	Q9QYR9	6.4	46	6.9	50	5	375	102	15
206	Elongation factor Tu	Q8BFR5	6.4	48	7.2	49	9	450	78	33
209	Isovaleryl-CoA dehydrogenase	Q9JHI5	6.6	44	8.5	46	14	513	131	44
212	Creatine kinase M-type	P07310	6.7	45	6.6	43	6	352	73	29
213	Isocitrate dehydrogenase	O88844	6.8	46	6.4	47	8	376	81	22
214	Beta-enolase	P21550	6.7	50	6.7	47	12	906	130	43
219	Mitochondrial inner membrane protein	Q8CAQ8	6.7	69	6.2	84	7	455	98	12
328	Catechol O-methyltransferase	O88587	5.0	29	5.5	29	1	28	28	4
331	Heat shock protein beta-1	P14602	5.8	22	6.1	23	6	283	75	32

Gene name and accession number according to SwissProt.Experimental (E) Isoelectric point (pI) and molecular weight (Mw). Theoretical (T) Isoelectric point (pI) and molecular weight (Mw).

Principal component analysis, performed on all spots detected is illustrated in Figure 3C and revealed patterns that clearly and orderly segregated the 12 groups. We have circled the sham-operated (S), noncongested (C) mice and mice with congestive heart failure (H) suggesting that the C group was intermediate between S and H.

#### Gender-related differential expression profile of mouse LV

Two-dimensional differential in-gel electrophoresis analysis revealed that 13 spots showed differential expression between male and female mice listed in Table 5. This comparison highlighted two proteins downregulated in males (Table 5), related to distinct functions, that is, xenobiotic detoxification (ESTN: liver carboxyl esterase N) and alcohol metabolism (ALDH2: aldehyde dehydrogenase, mitochondrial). Eleven spots (corresponding to eight proteins) were upregulated in male mice. Among these were all isoforms of alpha-1-antitrypsin 1 (A1AT1, A1AT2, A1AT3, and A1AT4), the fibrinogen beta chain (FIBB), protein disulfide-isomerase (PDIA1), and calreticulin (CALR).

**Table 5:** Gender comparison

					M versus F	
					
Spot number	Protein name	Uniprot accession	Accession number	1-ANOVA	*P*-value	FC
93	Calreticulin	CALR_MOUSE	P14211	2,9E-06	3,6E-11	2,9
96	Serine protease inhibitor A3K	SPA3K_MOUSE	P07759	2,4E-04	1,4E-06	1,8
101	Alpha-1 -antitrypsin 1-4	A1AT4_MOUSE	Q00897	9,5E-06	1,9E-09	3,2
102	Alpha-1-antitrypsin 1-3	A1AT3_MOUSE	Q00896	1,2E-06	5,8E-11	2,7
103	Alpha-1-antitrypsin 1-1	A1AT1_MOUSE	P07758	7,7E-08	1,3E-11	2,0
105	Alpha-1-antitrypsin 1-3	A1AT3_MOUSE	Q00896	4,0E-08	2,2E-12	1,8
106	Alpha-1-antitrypsin 1-1	A1AT1_MOUSE	P07758	3,6E-05	2,4E-09	1,7
107	Alpha-1-antitrypsin 1-2	A1AT2_MOUSE	P22599	6,0E-07	2,0E-08	2,0
110	Liver carboxylesterase N	EST1C_MOUSE	P23953	2,9E-01	8,2E-03	−1,7
182	Fibrinogen beta chain	FIBB_MOUSE	Q8K0E8	1,5E-04	2,0E-07	1,4
185	Aldehyde dehydrogenase, mitochondrial	ALDH2_MOUSE	P47738	2,5E-05	2,2E-04	−1,3
190	Aldehyde dehydrogenase, mitochondrial	ALDH2_MOUSE	P47738	5,0E-07	2,4E-05	−1,4
104	Protein disulfide-isomerase	PDIA1_MOUSE	P09103	5,8E-08	2,2E-04	1,4

FC, Fold change; using DeCyder software, between normalized spot volume between male (M) and female (F) mouse samples.

#### Genotype-related differential expression profile

Proteomic analysis revealed 11 identified proteins that were differentially expressed between wild type (W) and FKBP12.6-overexpressing mice (K) which are listed in Table 6. A set of differentially expressed proteins (14-3-3 G, ADK, GSTM5, GSTM7, ILEUA, KBTBA, MARE2, and SBP1 characterized the genotype difference [Table 6]). Among the upregulated proteins (Table 6), a marked upregulation of the detoxification enzymes, glutathione S-transferases (GSTM5 and GSTM7), was observed in K mice. SBP1, an antioxidant protein, was also upregulated in MKH mice. The cytoskeletal protein (MARE2: microtubule-associated protein RP/EB family member2) and the leukocyte elastase inhibitor (ILEUA) were downregulated in K mice whereas others such as PDZ and LIM domain protein1 (PDLI1 = PDLIM1) were simultaneously increased (Table 6).

**Table 6:** Genotype comparison

					K versus W	
					
Spot number	Protein name	Uniprot accession	Accession number	1-ANOVA	*P*-value	FC
9	Glutathione S-transferase Mu 7	GSTM7_MOUSE	Q80W21	1,2E-05	3,2E-04	1,4
10	Glutathione S-transferase Mu 5	GSTM5_MOUSE	P48774	2,4E-05	1,4E-08	4,0
17	Glutathione S-transferase Mu 5	GSTM5_MOUSE	P48774	5,1E-05	1,4E-08	5,4
31	14-3-3 protein gamma	1433G_MOUSE	P61982	2,5E-07	3,2E-04	1,2
47	Microtubule-associated protein RP/EB family member 2	MARE2_MOUSE	Q8R001	3,1E-06	3,8E-05	−1,3
53	PDZand LIM domain protein 1	PDLI1_MOUSE	070400	6,4E-06	1,5E-02	1,3
65	Leukocyte elastase inhibitor A	ILEUA_MOUSE	Q9D154	5,2E-05	8,9E-02	−1,4
85	Adenosine kinase	ADK_MOUSE	P55264	3,7E-07	3,2E-04	1,3
133	Kelch repeat and BTB domain-containing protein 10	KBTBA_MOUSE	Q9ER30	6,8E-05	6,6E-03	1,3
191	Sarcalumenin	SRCA_MOUSE	Q7TQ48	7,2E-04	3,2E-04	1,3
193	Selenium-binding protein 1	SBP1_MOUSE	P17563	4,0E-08	1,2E-03	1,3

FC, Fold change; using DeCyder software, between normalized spot volume between wild-type (W) and FKBP12.6-overexpressing (K) mouse samples.

#### Differential expression profiles of TAC-induced pathological LVH

**Figure 4:**
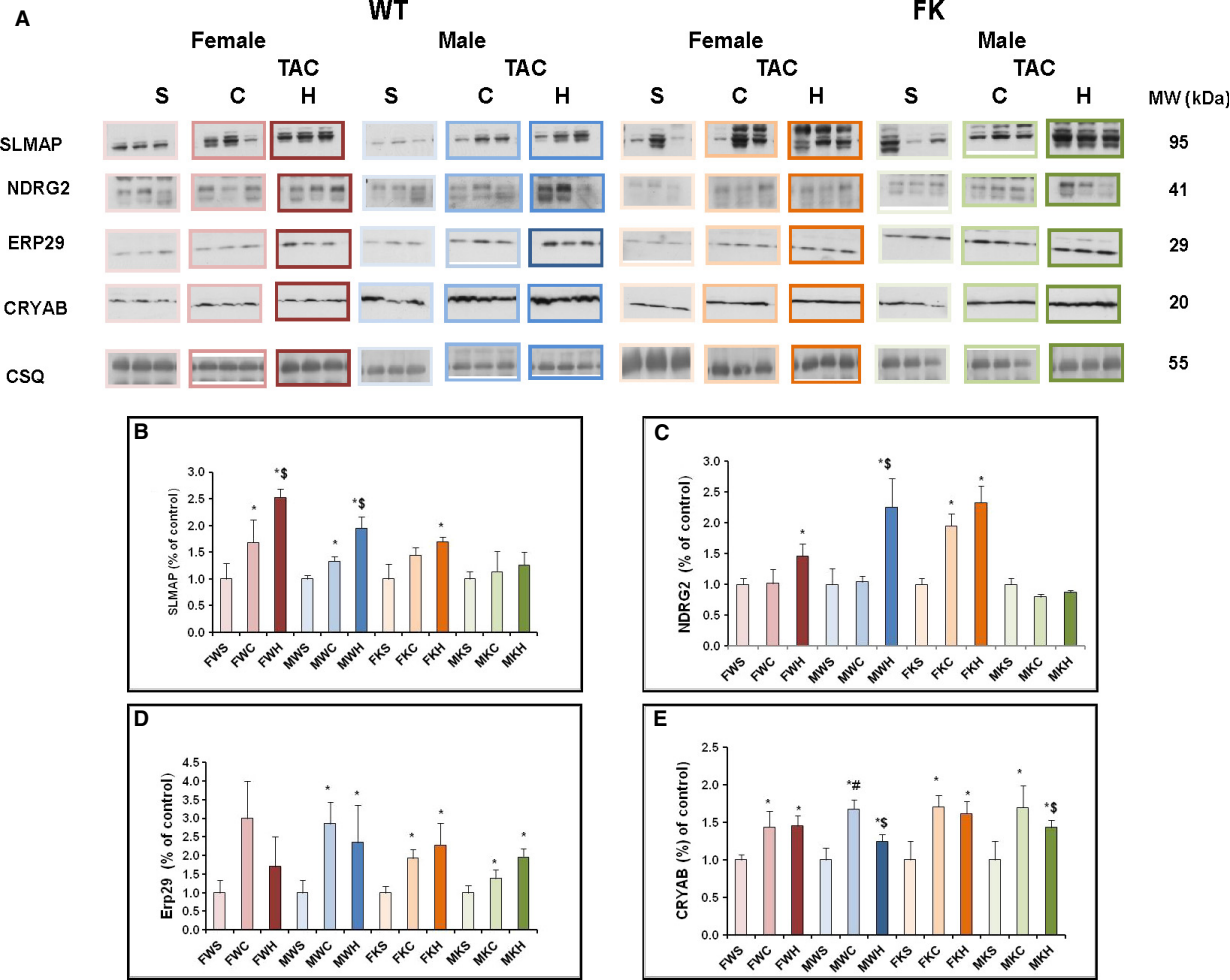
Validation of a few selected 2D DIGE proteins. (A), representative immunoblots of SLMAP, NDRG2, ERP29, and CRYAB expression levels in all experimental groups. Western blot of calsequestrin (CSQ) was used as a control of protein loading, and to normalize the densitometric data of each protein expression. (B–E), respective histograms of levels of SLMAP, NDRG2, ERP29, and CRYAB proteins (are expressed as fold) increases in TAC mouse groups (C and H) relative to their respective shams (S), in the FKBP12.6 overexpressing (K) group compared with wild-type mice (W) of both genders (M or F). Data are mean ± sem. *P < 0.05, TAC versus sham; $*P* < 0.05, C versus. H; #*P* < 0.05, female versus male.

At 30 days postsurgery, chronic PO induced LVH associated with an altered protein expression pattern (Table 7). We selected a few proteins differentially expressed and identified by 2D-DIGE for immunoblotting. Figure 4B–E show representative Western blot analyses of SLMAP (Fig. 4B), NDRG2 (Fig. 4C), endoplasmic reticulum stress-associated proteins (ERP29, Fig. 4D), and alpha-crystallinB chain (CRYAB, Fig. 4E) expression in C and H mice (from four individuals in each group) and CSQ as a control for protein loading (Fig. 4A). H mice exhibited an obvious upregulation of SLMAP and CRYAB. TAC induced a significant increase in NDRG2 protein level in MWH whereas no significant change was observed in MKH mice (Fig. 4C). TAC was also associated with an increased level of ERP 29 (Fig. 4D). These upregulations of the selected proteins were consistent with 2D-DIGE results.

**Table 7:** Differentially expressed and identified cardiac left ventricular spots in response to TAC in mice

					(C, H) versus S		C versus S		H versus S		H versus C	
								
Spot number	Protein name	Uniprot accession	Accession number	1-ANOVA	*P*-Value	FC	*P*-Value	FC	*P*-Value	FC	*P*-Value	FC
A: Spots with fold change not significantly different between H and C groups												
6	Ferritin heavy chain	FRIH_MOUSE	P09528	1,1E-02	6,9E-06	1,8	3,1E-03	1,8	7,6E-05	1,7	**5,3E-01**	**−1,0**
8	Alpha-crystallin B chain	CRYAB_MOUSE	P23927	5,6E-03	2,8E-05	2,0	2,2E-02	1,8	2,8E-05	2,2	**1,9E-01**	**1,3**
9	Glutathione S-transferase Mu 7	GSTM7_MOUSE	Q80W21	1,2E-05	1,3E-04	−1,4	1,2E-02	−1,3	1,4E-03	−1,4	**3,4E-01**	**−1,1**
18	Guanylate kinase	KGUA_MOUSE	Q64520	1,4E-05	3,4E-07	1,5	4,3E-03	1,4	4,4E-06	1,5	**2,3E-01**	**1,1**
23	Heat shock protein beta-1	HSPB1_MOUSE	P14602	6,4E-06	2,5E-06	2,1	5,9E-03	1,9	5,5E-06	2,3	**1,6E-01**	**1,2**
28	Heat shock protein beta-1	HSPB1_MOUSE	P14602	2,3E-03	6,0E-05	1,7	2,2E-02	1,5	8,1E-05	1,9	**1,4E-01**	**1,3**
43	Ubiquinone biosynthesis protein COQ9	COQ9_MOUSE	Q8K1Z0	1,1E-06	1,6E-06	−1,3	8,8E-03	−1,2	1,9E-06	−1,4	**4,5E-03**	**−1,1**
53	PDZ and LIM domain protein 1	PDLI1_MOUSE	070400	6,4E-06	5,8E-06	1,5	1,7E-02	1,4	5,0E-05	1,6	**1,3E-01**	**1,1**
70	L-lactate dehydrogenase B chain	LDHB_MOUSE	P16125	2,1E-04	6,3E-06	−1,3	1,4E-02	−1,2	1,6E-06	−1,3	**4,0E-02**	**−1,1**
71	Isocitrate dehydrogenase [NAD] subunit alpha	IDH3A_MOUSE	Q9D6R2	1,6E-04	1,6E-03	−1,2	5,7E-02	−1,2	2,1E-02	−1,2	**3,1E-01**	**1,1**
72	Protein ADP-ribosylarginine] hydrolase-like protein 1	ARHL1_MOUSE	Q8BGK2	1,2E-07	2,7E-08	1,4	3,8E-05	1,4	2,2E-06	1,4	**4,3E-01**	**−1,0**
75	L-lactate dehydrogenase B chain	LDHB_MOUSE	P16125	5,0E-03	2,8E-05	−1,4	4,4E-02	−1,3	7,0E-05	−1,5	**5,5E-02**	**−1,1**
85	Adenosine kinase	ADK_MOUSE	P55264	3,7E-07	6,0E-03	1,2	2,5E-01	1,2	1,0E-02	1,3	**3,0E-01**	**1,1**
119	Dynactin subunit 2	DCTN2_MOUSE	Q99KJ8	2,4E-03	2,2E-05	1,3	1,7E-02	1,3	1,2E-04	1,4	**1,7E-01**	**1,1**
128	Actin. alpha skeletal muscle	ACTS_MOUSE	P68134	4,8E-03	1,8E-03	1,2	2,4E-02	1,2	3,1E-03	1,3	**1,2E-01**	**1,1**
154	Serum albumin	ALBU_MOUSE	P07724	1,5E-04	2,0E-04	1,3	5,7E-02	1,3	1,4E-03	1,4	**3,3E-01**	**1,1**
185	Aldehyde dehydrogenase	ALDH2_MOUSE	P47738	2,5E-05	2,4E-03	−1,2	2,8E-01	−1,2	2,3E-03	−1,3	**1,3E-01**	**−1,1**
187	T-complex protein 1 subunit beta	TCPB_MOUSE	P80314	1,3E-02	3,7E-05	1,5	2,9E-02	1,4	6,7E-04	1,5	**4,2E-01**	**1,0**
193	Selenium-binding protein 1	SBP1_MOUSE	P17563	4,0E-08	5,8E-05	−1,4	6,4E-02	−1,3	1,1E-05	−1,5	**5,9E-02**	**−1,2**
209	Isovaleryl-CoA dehydrogenase	IVD_MOUSE	Q9JHI5	5,2E-04	6,3E-06	−1,3	3,8E-02	−1,2	7,0E-07	−1,4	**1,2E-01**	**−1,2**
B: Spots with fold change significantly different between H and C groups												
4	ATP synthase subunit delta	ATP5H_MOUSE	Q9DCX2	1,3E-02	5,2E-04	−1,4	**1,6E-01**	−**1,2**	3,3E-04	−1,6	5,0E-02	−1,3
7	Ferritin heavy chain	FRIH_MOUSE	P09528	3,9E-03	5,1E-05	−1,5	**5,7E-02**	−**1,3**	4,6E-06	−1,7	5,0E-02	−1,3
12	Long-chain specific acyl-CoA dehydrogenase	ACADL_MOUSE	P51174	9,3E-04	1,6E-04	−1,3	**1,4E-01**	−**1,2**	2,5E-05	−1,4	2,9E-02	−1,2
20	Thioredoxin-dependent peroxide reductase	PRDX3_MOUSE	P20108	6,4E-06	2,6E-06	−1,4	1,9E-02	−1,2	2,5E-08	−1,5	3,9E-03	−1,3
21	Endoplasmic reticulum resident protein 29	ERP29_MOUSE	P57759	5,3E-03	2,9E-04	1,4	**7,4E-02**	**1,2**	1,3E-04	1,6	2,7E-02	
30	14-3-3 protein beta/alpha	1433B_MOUSE	Q9CQV8	3,2E-04	2,9E-04	1,2	**2,7E-01**	**1,1**	2,7E-06	1,3	6,0E-04	1,2
32	14-3-3 protein zeta/delta	1433Z_MOUSE	P63101	2,3E-05	2,8E-05	1,2	**6,0E-02**	**1,1**	2,4E-07	1,3	1,1E-03	1,2
35	Tropomyosin alpha-3 chain	TPM3_MOUSE	P21107	1,4E-04	1,7E-05	1,4	**5,9E-02**	**1,2**	1,1E-08	1,6	3,7E-03	1,3
39	Annexin A5	ANXA5_MOUSE	P48036	6,0E-07	1,5E-07	1,4	3,8E-03	1,3	6,2E-09	1,6	1,6E-04	1,3
44	Chloride intracelular channel protein 1	CLIC1_MOUSE	Q9Z1 Q5	5,6E-04	1,4E-05	1,3	**6,2E-02**	**1,2**	8,1E-07	1,4	1,5E-03	1,2
45	Sarcolemmal membrane-associated protein	SLMAP_MOUSE	Q3URD3	2,5E-07	3,7E-08	1,6	1,2E-03	1,4	2,8E-08	1,8	1,1E-03	
46	Sarcolemmal membrane-associated protein	SLMAP_MOUSE	Q3URD3	5,8E-08	1,5E-09	1,8	2,9E-04	1,6	5,8E-09	2,0	7,2E-04	1,3
48	F-actin-capping protein subunit beta	CAPZB_MOUSE	P47757	7,7E-08	5,6E-07	1,4	8,8E-03	1,3	1,0E-08	1,6	6,3E-03	1,2
51	Electron transfer flavoprotein subunit alpha	ETFA_MOUSE	Q99LC5	1,9E-03	4,3E-05	−1,3	**7,1E-02**	**−1,2**	4,6E-05	−1,4	1,9E-02	−1,2
58	Delta (3,5)-Delta (2,4)-dienoyl-CoAisomerase	ECH1_MOUSE	035459	4,0E-08	3,7E-08	−1,7	1,8E-03	−1,4	2,3E-10	−2,0	3,1E-04	−1,4
60	Delta (3,5)-Delta (2,4)-dienoyl-CoAisomerase	ECH1_MOUSE	035459	5,8E-08	1,1E-07	−1,7	3,9E-03	−1,5	2,2E-08	−2,0	7,0E-04	−1,3
62	Malate dehydrogenase	MDHC_MOUSE	P14152	2,3E-03	1,8E-05	−1,3	3,6E-02	−1,2	9,8E-06	−1,4	2,7E-02	−1,2
63	Trans-1,2-dihydrobenzene-1,2-diol dehydrogenase	DHDH_MOUSE	Q9DBB8	2,0E-02	1,1E-03	−1,3	**1,9E-01**	**−1,2**	6,7E-04	−1,5	2,3E-02	−1,3
65	Leukocyte elastase inhibitor A	ILEUA_MOUSE	Q9D154	5,2E-05	1,4E-02	1,4	**6,5E-01**	**1,1**	1,5E-03	1,8	1,8E-02	−1,6
74	Serpin B6	SPB6_MOUSE	Q60854	6,4E-06	2,8E-06	1,4	1,8E-02	1,2	1,3E-06	1,5	1,7E-03	1,2
78	Isocitrate dehydrogenase [NAD] subunit alpha	IDH3A_MOUSE	Q9D6R2	1,1E-05	1,8E-03	−1,3	**5,9E-01**	**−1,1**	2,7E-06	−1,6	6,2E-04	−1,5
89	Protein NDRG2	NDRG2_MOUSE	Q9QYG0	4,1E-03	1,1E-02	1,3	**6,9E-01**	**1,1**	1,7E-03	1,5	1,8E-02	1,4
104	Protein disulfide-isomerase	PDIA1_MOUSE	P09103	5,8E-08	3,1E-04	1,4	**1,8E-01**	**1,2**	4,5E-05	1,5	9,7E-03	1,3
115	ATP synthase subunit beta	ATPB_MOUSE	P56480	1,5E-03	6,7E-06	−1,4	1,7E-02	−1,3	2,5E-05	−1,5	2,9E-02	-1,2
116	ATP synthase subunit beta	ATPB_MOUSE	P56480	2,6E-05	2,8E-07	−1,4	5,0E-03	−1,3	2,4E-07	−1,5	1,6E-03	-1,2
120	Desmin	DESM_MOUSE	P31001	2,7E-05	2,8E-05	1,4	6,7E-02	1,2	4,5E-08	1,6	7,4E-04	1,4
121	60 kDa heat shock protein	CH60_MOUSE	P63038	2,5E-07	5,7E-05	−1,2	**1,4E-01**	**−1,1**	8,3E-09	−1,3	5,3E-06	−1,2
122	60 kDa heat shock protein	CH60_MOUSE	P63038	5,2E-05	1,5E-05	−1,3	**6,1E-02**	**−1,2**	1,7E-07	−1,5	2,5E-04	−1,3
123	Vimentin	VIME_MOUSE	P20152	3,1E-07	7,1E-07	1,6	4,3E-03	1,4	7,1E-10	1,9	9,2E-04	1,4
124	Desmin	DESM_MOUSE	P31001	1,5E-04	8,8E-05	1,4	**1,2E-01**	**1,2**	1,2E-07	1,6	1,6E-03	1,4
125	Vimentin	VIME_MOUSE	P20152	3,3E-06	4,7E-06	1,5	1,7E-02	1,3	2,1E-08	1,7	1,1E-03	1,4
126	Vimentin	VIME_MOUSE	P20152	4,9E-06	3,0E-06	1,4	1,4E-02	1,2	1,7E-08	1,5	3,0E-03	1,3
127	Vimentin	VIME_MOUSE	P20152	1,9E-04	5,8E-05	1,4	**1,2E-01**	**1,2**	2,4E-07	1,6	2,0E-03	1,3
130	NADH-ubiquinone oxidoreductase 75 kDa subunit	NDUS1_MOUSE	Q91VD9	5,2E-04	1,6E-04	−1,3	**1,8E-01**	**−1,2**	2,8E-05	−1,5	7,0E-04	−1,3
132	NADH-ubiquinone oxidoreductase 75 kDa subunit	NDUS1_MOUSE	Q91VD9	4,8E-03	1,3E-04	−1,3	**1,4E-01**	**−1,2**	5,0E-05	−1,5	4,1E-03	−1,2
133	Kelch repeat and BTB domain-containing protein 10	KBTBA_MOUSE	Q9ER30	6,8E-05	2,4E-04	1,3	**1,4E-01**	**1,2**	3,4E-04	1,5	2,5E-02	1,2
134	Kelch repeat and BTB domain-containing protein 10	KBTBA_MOUSE	Q9ER30	2,2E-04	5,8E-05	1,2	**7,3E-02**	**1,2**	1,8E-05	1,3	1,5E-02	1,2
135	Protein-glutamine gamma-glutamyltransferase 2	TGM2_MOUSE	P21981	9,6E-06	2,8E-07	1,8	2,8E-03	1,5	3,5E-08	2,0	8,8E-03	1,4
136	Protein-glutamine gamma-glutamyltransferase 2	TGM2_MOUSE	P21981	6,6E-06	6,9E-07	1,6	2,8E-03	1,4	2,4E-07	1,9	7,7E-03	1,3
143	Collagen alpha-l (VI) chain	C06A1_MOUSE	Q04857	7,4E-05	1,4E-05	1,7	**5,7E-02**	**1,4**	1,2E-07	2,0	2,0E-03	1,5
144	Collagen alpha-1 (VI) chain	C06A1_MOUSE	Q04857	3,6E-03	**6,2E-02**	**1,6**	**2,3E-01**	**1,3**	7,6E-05	2,0	9,7E-03	1,5
148	Major vault protein	MVP_MOUSE	Q9EQK5	2,8E-07	1,5E-09	1,7	3,8E-05	1,5	2,1E-09	1,8	2,8E-02	1,2
168	Moesin	MOES_MOUSE	P26041	5,3E-04	1,7E-03	1,3	**3,5E-01**	**1,1**	1,4E-04	1,5	4,1E-03	1,4
169	Serotransferrin	TRFE_MOUSE	Q921I1	3,4E-02	3,9E-02	1,2	**9,1E-01**	**1,0**	1,1E-02	1,3	2,8E-02	1,3
170	Moesin	MOES_MOUSE	P26041	1,5E-02	7,8E-04	1,3	**2,6E-01**	**1,1**	4,4E-05	1,4	6,8E-03	1,3
173	Moesin	MOES_MOUSE	P26041	4,5E-03	1,2E-03	1,4	**3,5E-01**	**1,2**	1,0E-05	1,6	4,3E-03	1,4
177	Propionyl-CoA carboxylase beta chain, mitochondrial	PCCB_MOUSE	Q99MN9	5,8E-08	1,5E-07	−1,9	4,2E-03	−1,5	5,9E-10	−2,3	2,3E-03	−1,5
179	Carboxylesterase 1D	CES3_MOUSE	Q8VCT4	1,2E-07	6,9E-06	−2,0	5,1E-02	−1,6	1,7E-06	−2,5	4,0E-05	−1,6
184	Dihydropyrimidinase-related protein 2	DPYL2_MOUSE	008553	1,9E-04	1,5E-05	1,3	4,2E-02	1,2	9,6E-07	1,4	2,9E-03	1,2
186	Lipoamide acyltransferase component of branched-chain alpha-keto acid dehydrogenase complex	ODB2_MOUSE	P53395	9,1E-06	3,4E-07	−1,3	8,9E-03	−1,2	2,3E-10	−1,5	7,2E-03	−1,2
188	Adenylyl cyclase-associated protein 2	CAP2_MOUSE	Q9CYT6	4,5E-04	1,7E-04	1,4	**1,6E-01**	**1,2**	1,7E-04	1,7	6,2E-04	1,4
197	EH domain-containing protein 4	EHD4_MOUSE	Q9EQP2	1,4E-04	4,8E-02	1,2	**8,7E-01**	**1,0**	6,7E-04	1,4	8,3E-03	1,4
199	Septin-2	SEPT2_MOUSE	P42208	1,1E-05	6,9E-06	1,5	2,5E-02	1,3	3,5E-08	1,7	1,1E-03	1,4
203	Short/branched chain specific acyl-CoA dehydrogenase	ACDSB_MOUSE	Q9DBL1	1,3E-03	5,4E-03	−1,2	**3,3E-01**	**−1,1**	8,6E-03	−1,3	2,3E-02	−1,2
204	Acyl-coenzyme Athioesterase 2	ACOT2_MOUSE	Q9QYR9	3,1E-05	1,6E-05	−1,3	4,4E-02	−1,2	2,4E-08	−1,6	4,0E-04	−1,3
205	Acyl-coenzyme Athioesterase 2	ACOT2_MOUSE	Q9QYR9	5,3E-05	2,1E-05	−1,3	3,6E-02	−1,2	2,4E-07	−1,5	6,2E-04	−1,3
206	Elongation factor Tu	EFTU_MOUSE	Q8BFR5	1,1E-02	2,7E-03	−1,3	**5,6E-01**	**−1,1**	7,5E-05	−1,6	1,1E-03	−1,5
212	Creatine kinase M-type	KCRM_MOUSE	P07310	9,3E-04	1,6E-04	−1,3	**1,4E-01**	**−1,2**	2,5E-05	−1,4	2,9E-02	−1,2
213	Isocitrate dehydrogenase [NADP]	IDHC_MOUSE	088844	7,2E-03	4,8E-03	−1,3	**5,5E-01**	**−1,1**	2,0E-04	−1,5	5,9E-03	−1,4

S, sham-operated mice; (C, H), TAC mice; C, noncongested mice; H, mice with congestive heart failure; FC, fold change; bold values, fold change not statistically valid (*P* > 0.05).

#### Bioinformatic analysis of proteomic finding

**Figure 5:**
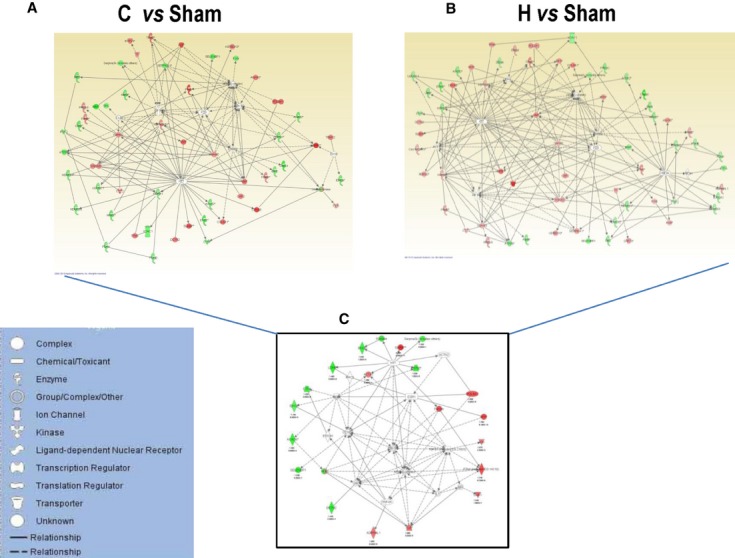
TAC-induced specific molecular signatures. Graphical representation of the most significant protein interaction networks using ingenuity pathway analysis (IPA) of differentially expressed identified proteins. The upregulated proteins are marked in red and the downregulated in green. The nodes represent proteins that are connected with one or several arrows; the solid arrows represent direct interactions and the dotted arrows indirect interactions. (A), the network shows the difference between noncongested (C) and nonfailing hearts (S), (B), the network demonstrates the difference between congestive failing hearts (H) and nonfailing hearts (S), (see Table 7). (C) Common bio-signature of TAC-induced pathological LVH: an IPA analysis shows that the TAC induces similar alterations in protein content in C and H mice. Upregulated identified proteins are depicted in red and downregulated identified proteins in green. Solid lines indicate direct interaction or regulation, and dashed lines indirect relationships.

**Figure 6:**
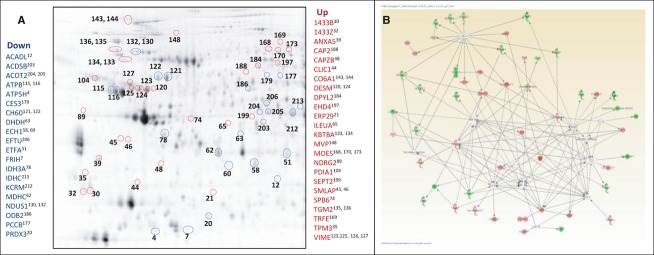
Comparative differentially expressed and identified protein spots (H vs. C). (A) 2D-DIGE analysis revealed 59 spots differentially expressed in HF analysis. Thirty-four spots were upregulated (surrounded in red) and 25 spots were downregulated (surrounded in blue) in the FKBP12.6 overexpressing mouse group compared with wild-type mice. (B) The networks demonstrate the difference between congestive failing hearts (H) and noncongestive hearts (C), upregulated (red), and downregulated proteins (green) where direct interactions or regulation (solid lines), and indirect relationships (dashed lines) are shown. (C). Here is the list of differentially expressed identified spot proteins in H mice.

Ingenuity pathway analysis allowed us to place the differential proteomic findings into a biological context. After giving information concerning their location within the cell, identified proteins are subdivided into slices based on cellular functions (Table 7) or involvement in canonical pathways. The building of networks shows how an identified molecule can participate in a given biological pathway. According to the presence of pulmonary edema, mice with congestive failing hearts (H) or without lung congestion (noncongested mice, C) were compared with their respective sham-operated mice (S). Figures 5 and 6 show the IPA networks obtained from data related to the following two TAC-induced differential protein expression profiles; first, between C mice and S mice (Fig. 5A) and second, between H mice and S mice (Fig. 5B).

When H mice were compared with C mice, they exhibited a specific molecular signature (Fig. 6A and B) not found in C mice.

##### Similarities of PO-induced changes in C and H mice

Compared to sham mice, 26 identified spots were differentially expressed in approximatively the same range for C and H mice, and were not significantly different between H and C groups. Out of these 26 spots, 12 spots were upregulated and eight spots were downregulated in TAC mice (Table 7A). In TAC mice, a similar expression pattern (Fig. 5C) was observed for proteins related to the electron transport chain (ETFA: electron transfer flavoprotein subunit alpha; COQ9: ubiquinone biosynthesis protein COQ9), and to the citric acid cycle (IDH3A). Most of these key enzymes displayed various degrees of downregulation. As expected, alpha skeletal actin (ACTS) was upregulated in TAC mice. Serum albumin was also increased in TAC mice. A significant upregulation of major vault protein (MVP, also known as lung resistance-related protein), SLMAP, PDZ and LIM domain protein1 (PDLI1 = PDLIM1) was observed in all TAC mice.

##### Molecular signature of TAC-induced congestive failing mouse heart (H)

Interestingly, within the group of TAC mice, the molecular signature of those with congestive HF (H) differed from that of mice without pulmonary edema (C) (Fig. 5A–B). Indeed, a different expression pattern was seen between the two phenotypes. Of 59 differentially expressed proteins, 34 were upregulated and 23 were downregulated in H mice relative to C mice (Fig. 6A and Table 7B). Decreases in ferritin heavy chain (FRIH = FTH1), 60 kDa heat shock protein (CH60 = HSPD1), and peroxiredoxin 3 (PRDX3) were observed. The downregulation in the mitochondrial proteins (Acyl-coenzyme A thioesterase2 [ACOT2], carboxylesterase 3 [CES3], a component of branched-chain alpha-keto acid dehydrogenase [ODB2], propionyl-CoA carboxyl beta chain [PCCB]) associated with metabolic energy processor playing a role in the tricarboxylic acid cycle (isocitrate dehydrogenase: IDHC) or the respiratory chain (ATP synthase subunit d: ATP5H) were also observed.

Most of the protein upregulations occurring in H mice concerned cytoskeletal remodeling (F-actin-capping protein subunit beta: CAPZB; desmin [DSM = DES]; dihydropyrimidinase-related protein2 [DPYL2]; microtubule-associated protein RP/EB family member2 [MARE2]; moesin [MOES = MSN]; septine 2 [SEPT2]; tropomyosin alpha3-chain [TPM3]; vimentin [VIM]). Moreover, proteins involved in antioxidative mechanisms were also upregulated in H mice. In addition, the stress-responsive glutathione S-transferase omega-1 (GSTO-1) and proteins of calcium homeostasis, like annexin A5 (ANXA5), were also higher in congestive failing hearts. Protein-glutamine gamma-glutamyltransferase 2 (TGM2), known to interact with a number of different substrates and to play a role in the response to injury, is associated with congestive HF. On the one hand, the intracellular serpin (leukocyte elastase inhibitor A [ILEUA]) protein level was higher in H than in C mice; on the other hand, we found an increase in serpin B6 (SPB6). In addition, several proteins: adenylyl cyclase–associated protein2 (Cap2), chloride intracellular channel protein1 (CLIC1), EH domain-containing protein 4 (EHD4), Kelch repeat and BTB domain-containing protein 10 (KBTBA), NDRG2 and endoplasmic reticulum protein ERp29 (ERP29) were upregulated in failing LVs.

#### Validation of cardiac HTT expression and one of its direct interactions by immunoblotting

**Figure 7:**
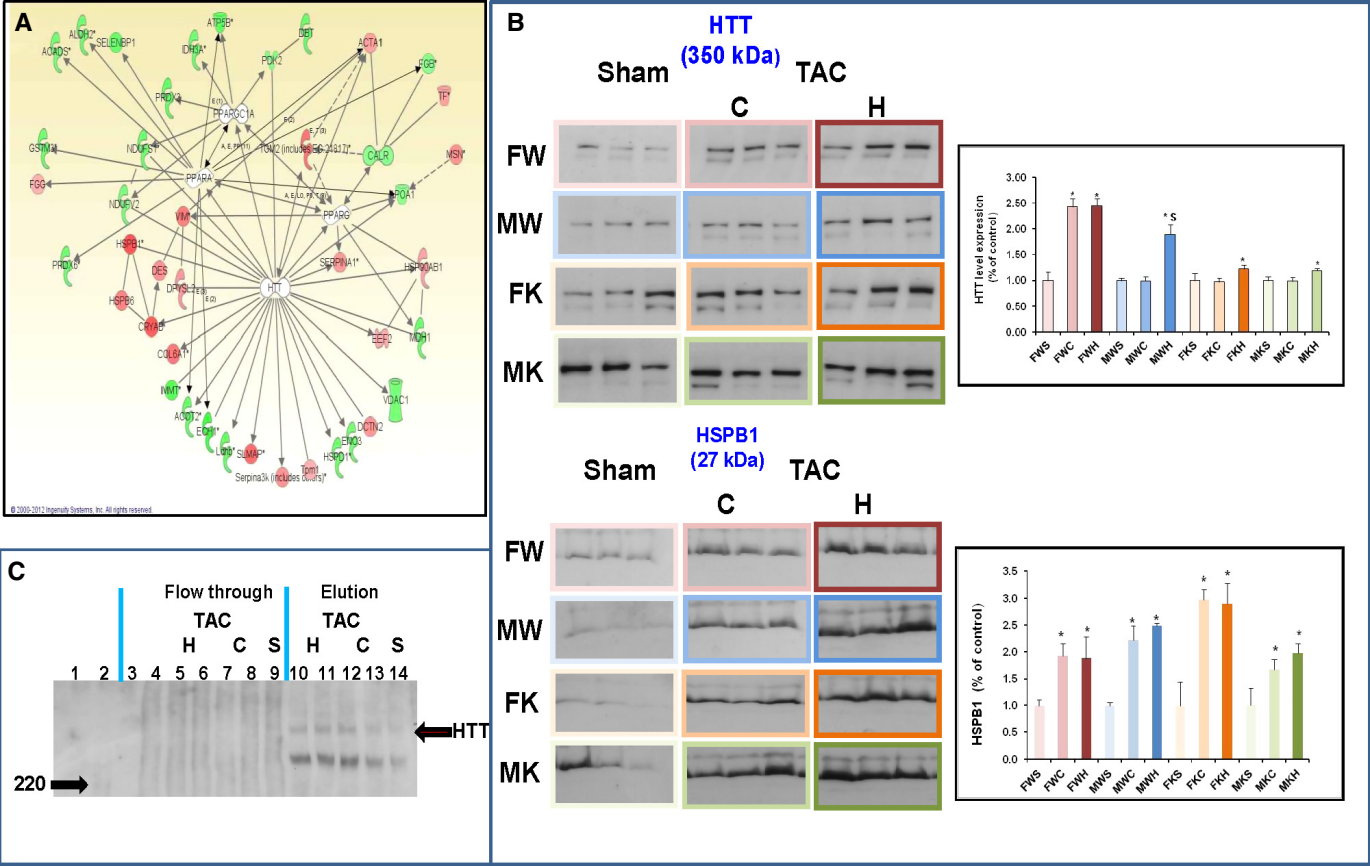
Predictive emerging role of huntingtin in heart failure. (A) In order to predict how the cross-talk among proteins takes part in the progression of heart failure, by using IPA software we organized identified proteins in congestive failing heart (H) into different networks. It appears that one major network, in which huntingtin protein (HTT) was found, gathers proteins involved in mitochondrial function and cytoskeletal remodeling. (B) Western blotting analysis validates the expression of HTT and HSPB1 in mouse heart of both strains after TAC. (C). Coimmunoprecipitation of HTT with endogenous HSPB1 (Hsp27). The anti-Hsp27 antibody was used to immunoprecipitate HSPB1 from mouse heart lysates. Immunoprecipitates (IP) separated by SDS-PAGE electrophoresis (3–8% Tris-Acetate) and probed with an anti-HTT. *Lane 1*, brain lysate; *lane 2*, MagicMark™ XP Western protein standard in the range of 20–220 kDa; *lane 3*, HSP27 immunoprecipitate with Protein A/G sepharose magnetic beads in the absence of lysate; *lane 4*, lysate precipitated with Protein A/G beads lacking anti-Hsp27 antibody; *lanes: 5-9*, respective flow-through of LV lysates immunoprecipitated with anti-HSP27*; lanes10-14*, eluates of LV lysates immunoprecipitated with anti-HSP27 linked to Protein A/G sepharose (*lanes 10-11*, IP from two individual H mouse LV; *lanes12-13*, IP from two individual C mouse LV; *lane 14*, IP from S mouse LV). HTT was coimmunoprecipitated with endogenous HSPB1 in cardiac lysates.

Ingenuity pathway analysis revealed that HTT is one of the most interconnected nodes involving 23 differentially identified proteins in C mice (Fig. 5A) and related directly or indirectly with still more proteins in H mice (Fig. 5B). In order to estimate the predictive involvement of HTT in HF (Fig. 7A), the Western blot analysis illustrated in Figure 7B shows that TAC is associated with a significant increase in the expression level of HTT in H mice whatever the gender or genotype. Here, we showed that the HSPB1 protein expression level was upregulated in TAC mice. These results were consistent with those of 2D-DIGE. Coimmunoprecipitation experiments (with the Hsp27 antibody) show a physical interaction between HSPB1 and HTT in mouse hearts (Fig. 7C), which confirmed the IPA predictive direct interaction HTT/HSPB1.

## Discussion

The present study deals with the integrative physiology of the effects of TAC at day 30 in mice overexpressing FKBP12.6 and their wild-type controls of both genders. As expected, TAC induced LVH. Despite no difference in stenotic jets indicating a similar degree of aortic constriction in all TAC mice studied, two distinct TAC-induced LVH responses were observed: without lung pathology, considered as likely compensatory LVH, (C) or with pulmonary edema, considered as congestive heart failure (H). Following TAC, mice of both genotypes exhibited similar systolic and diastolic LV dysfunction. These observations were associated with pronounced changes in the pattern of protein expression. The proteomic approaches used here allowed separation of proteins only within the range of approximately 10–150 kDa and between pH ranges of 4–7. Therefore, we were able to analyze here a substantial part of, but not the entire, LV proteome.

The major new findings are that (1) gender differences exist in plasma inflammatory response proteins; (2) there are genotype differences in the detoxifying enzymes; (3) differential comparative proteomic and bioinformatic analysis allowed us to distinguish molecular changes occurring in both C and H mice as a bio-signature of the early stage of failure initiation, then drastic changes in the abundance of mitochondrial proteins and antioxidant stress proteins in H mice that could be a signature characterizing the late end stage of HF; 4) according to the networks built using the IPA software, pathway analysis identified the HTT signaling node as a potential mediator of mitochondrial changes (Fig. 7) upon TAC-induced congestive HF.

### Gender molecular signature

In humans and mice, several groups reported that males exhibit higher α1-antitrypsin expression than females (Zabel et al. 2002; Regitz-Zagrosek 2006; Diedrich et al. 2007). Consistent with these previous reports, although serum contaminations of our myocardial LV samples cannot be totally excluded, we found that the cardiac protein level of the four isoforms of alpha1-antitrypsin and fibrinogen beta chain were more highly expressed in male than in female mice, whether submitted to TAC or not. Cardiac α1-antitrypsin expression might confer multiple protective roles associated with its antiinflammatory and immunomodulatory properties (Janciauskiene et al. 2011). Calreticulin, a multifunctional Ca^2+^-buffering chaperone, is involved in multiple cell processes (Michalak et al. 1999; Papp et al. 2009). TAC increased its expression in both genders and genotypes and male mouse hearts of both genotypes expressed higher calreticulin levels than female hearts. Mice overexpressing cardiac calreticulin exhibited a decreased systolic function and chamber dilation (Nakamura et al. 2001). Accordingly, it can be suggested that the TAC-induced upregulation of calreticulin plays a role in systolic function in our mouse model.

It is generally accepted that the mitochondria can serve as a source of NO-based cell signals that may originate independently of NO synthase activity (Chen et al. 2005). Mitochondrial ALDH2 (mtALDH2) is the main enzyme involved in acetaldehyde oxidation and in NO formation (Daiber et al. 2009; Song et al. 2011). The cardioprotective role of mtALDH2 is well known from studies in knockout mice (ALDH2^-/-^ mice), who show marked vascular dysfunction (Wenzel et al. 2008). In addition, it is tempting to speculate that the lower cardiac expression of mtADLH2, found here in males, might result in less NO production. There is ample evidence that in PO-induced LVH and end-stage HF, the substrate preference switches from free fatty acids (FFA) to glucose and is associated with a downregulation of enzymes involved in FFA metabolism (Ventura-Clapier et al. 2002; Mettauer et al. 2006; Regitz-Zagrosek et al. 2010). Liver carboxylesterase N (ESTN) although predominantly expressed in liver, is also expressed at lower levels in the heart (Islam et al. 1999; Diczfalusy et al. 2001). We found lower levels of ESTN in male than in female hearts, indicative of a gender difference in fatty acid metabolism.

Given all these concomitant changes, male and female mouse hearts differed in terms of Ca^2+^-homeostasis, energy metabolism, signaling, and stress responses, but the outcome of such differences still remains unclear. Furthermore, in whole LV tissue it is difficult to define whether all differential protein expression occurred exclusively in the cardiomyocytes, as other cell types could also contribute.

### Genotype molecular signature

Mice overexpressing FKBP12.6 differed markedly from wild-type mice; they exhibited very high levels of detoxification enzymes such as glutathione S-transferases (GST Mu5 and GST Mu7) and SBP1. Based on these findings, we may speculate that K mice handle oxidative stress differently, compared with WT mice. Interestingly, we found higher adenosine kinase (ADK) expression in K mouse hearts. ADK, a key enzyme in the purine salvage pathway, prevents toxic levels of adenosine building up within the cell (Kulkarni et al. 1998). This suggests that transgenic mice may also manage the adenosine pathway differently. We also observed a genotype difference in PDZ and LIM domain protein, with a small upregulation in K mice. These proteins may act as adapters between kinases and the cytoskeleton (Kotaka et al. 2001; Kadrmas and Beckele 2004) and colocalize with α-actinin at sites of actin anchorage, such as the intercalated disks of cardiac muscle cells. In this context, we can speculate that K mice may also present differences in the regulation of actin structure and dynamics. Besides this, MAPRE2 is one of the three microtubule-associated proteins that regulate microtubule functions and dynamics (Abiatari et al. 2009). Thus, the downregulation of MAPRE2 levels in K mice might alter the dynamics of cell morphology. However, addressing the functions of MAPRE2 in the heart awaits the generation of a mouse knockout model.

### TAC induced two molecular bio-signatures of pathological LVH

Thoracic aortic constriction causes chronic PO leading to pathological LVH and its progression to HF is accompanied with increases in cell size and surface area, and in protein synthesis. TAC also induced pulmonary edema in a subgroup of mice (H) of both genders and genotypes. Both C and H mice shared similar TAC-induced changes in a set of common proteins, either up- or downregulated, indicative of molecular events occurring at the early stage of HF.

### Molecular bio-signature of the early stage of progression to HF

We found that TAC induced an increase in two essential enzymes involved in purine metabolism, that is, ADK and KGUA (GUK1), each exerting its action through different cell signaling pathways in a dependent manner via their cellular localization. More recently, it was shown that ADK acts as an important mediator of adenosine attenuation of cardiomyocyte hypertrophy (Fassett et al. 2011) and also as a homeostatic bioenergetic network regulator adenosine (Shen et al. 2011). Here, TAC induced higher ADK protein levels in K hearts than in respective controls, and it is tempting to speculate that ADK might be a potential target for modulating the cardiac injury level in the early stage of HF in K mice. Besides these, KGUA catalyzes the phosphorylation of GMP to GDP and is also implicated in the regulation of the supply of guanine nucleotide to cell signaling pathways. Moreover, GMP-induced KGUA conformational changes are sufficient to convert KGUA to a membrane-associated guanylate kinase (MAGUK) involved in stabilizing cell–cell adhesion (Johnston et al. 2011). These protein dynamics raise the question of whether such neofunctionalization can occur in vivo in PO hearts. However, it is still unclear how the heart manages the balance between adenosine and guanine nucleotide availability.

Here, the MVP level was upregulated in C mice and further increased in failing mice (H). Despite the diverse conditions characterized by the upregulation of MVP, such as chemotherapy resistance (Scheffer et al. 2000), new roles have been recently assigned to MVP, including the association with insulin-like growth factor-1, hypoxia-inducible factor-1 alpha, and the major DNA repair machineries (Lara et al. 2001). Although the function of MVP is still largely unknown in the heart, in view of our observations, we may consider MVP as a potential prognostic factor associated with the progression to HF.

We have also identified two isoforms of SLMAP in mouse hearts of both genotypes. SLMAP plays a potential role in organizing the excitation–contraction (E–C) coupling apparatus of the cardiomyocyte and resides at distinct subcellular locations (Guzzo et al. 2005). Mice overexpressing cardiac SLMAP exhibit less response to an isoproterenol challenge, and their altered cardiac function was associated with diminished expression of Ca^2+^-handling proteins of SR, such as RyR2 and SERCA2a (Nader et al. 2012). Together these observations and our previous report showing downregulated Ca^2+^-handling proteins (Prévilon et al. 2011), suggest that the presently observed TAC-induced SLMAP upregulation plays a potential role in the impaired systolic and diastolic function in mouse hearts of both genotypes. As SLMAP level was higher in congestive failing hearts, it might be associated with diastolic dysfunction. We may suggest that SLMAP could qualify as a biomarker for the transition to HF.

Among the downregulated proteins observed in TAC mouse hearts of both genotypes were two glycolytic enzymes (ENOB [beta-enolase] and LDHB [L-lactate dehydrogenase B chain]). A decrease in ENOB was previously reported in rat pressure-overloaded hearts (Keller et al. 1995). Besides their glycolytic functions, they have also been shown to be suitable auxiliary proteins for DNA biology (Popanda et al. 1998). Concomitant alterations in given metabolic pathways were reflected by net changes in enzymes leading to mitochondrial dysfunction. Together, altered ACADS and ETFA levels involved in mitochondrial fatty acid beta-oxidation, and ATPB in the proton transport during oxidative phosphorylation, might contribute to decrease oxidative production of ATP, but also potentially increase production of reactive oxygen species (ROS). The decrease in IDH3A would impair the tricarboxylic acid cycle and the decrease in ubiquinone biosynthesis protein COQ9 would ensure a deficit in lipophilic antioxidant and therefore may affect the respiratory electron transfer chain. Taken together, these observations confirmed that mitochondrial machinery is altered in TAC-induced pathological LVH. Multiple changes in mitochondrial oxidative stress protein expression are determinant for the progression to HF whatever the genotype. Indeed, HF is frequently associated with energetic impairment (De Sousa et al. 2002; Neubauer 2007; Ingwall 2009; Turer et al. 2010).

### Molecular bio-signature of the end stage of HF

Multiple pathological changes have been reported at different levels in failing hearts. The impairment of EF and the increase in LVEDD, reflecting chamber dilatation, are key changes at the organ level.

At the tissue level, our previous work showed less TAC-induced LV fibrotic response in K mice, with a predominant perivascular but also interstitial fibrosis (Prévilon et al. 2011), potentially impairing oxygen diffusion. Here, the noticeably enhanced collagen type 6 (Co6A) expression level in MWH may explain, at least in part, diastolic dysfunction whereas MKT mice with a preserved diastolic function exhibited a smaller increase in CO6A. Thus, it is conceivable that the fibrotic response may result in a chronic hypoxic stress in congestive failing hearts. In agreement with recent data showing cardiomyocyte NDRG2 expression (Sun et al. 2011) and upregulation by hypoxia-induced stress (Wang et al. 2008), the present upregulation of NDRG2 might be attributed to the hypoxic environment. Recently, its adenoviral-mediated overexpression attenuated liver fibrosis (Yang et al. 2011). Elucidation of the biological function of NDRG2 in HF may provide a promising strategy for the treatment of myocardial fibrosis. Protein–glutamine gamma-glutamyl transferase (TGM2) functions as an extracellular matrix stabilizer (Deasey et al. 2013) and its Ca^2+^-dependent cross-linking activity has been implicated in many fibrotic diseases (Tovar-Vidales et al. 2011). Therefore, we can speculate that enhanced TGM2 expression levels participate in TAC-induced cardiac fibrosis although additional experimental studies are needed to assess its precise involvement in HF.

At the cell level, changes in cytoskeletal structure in LVH and HF have also been reported (Periasamy et al. 2008; Dhalla et al. 2009; Palazzuoli and Nuti 2010). Here, we observed the upregulation of several cytoskeletal proteins which might contribute to organize a network of proteins combining structural and signaling functions. Among them, desmin is well known to provide connections between the different organelles (nucleus, mitochondrion, sarcolemma) and influences their localization and function. Desmin is increased in human failing hearts and causes a loss of myocyte cross-striation (Heling et al. 2000). The increment in desmin observed here in H mice may also result from changes in function of its chaperone molecule, αB-crystallin (CryAB). Recently, molecular chaperones or heat shock proteins (HSPB6, HSPB1, CryAB) have been considered as multifunctional protective agents, their actions being implicated in intracellular protein quality control (QPR) (Kumarapeli et al. 2010) and in maintaining muscle integrity (Edwards et al. 2011) and contractile function (Fan and Kranias 2011). Besides these cardioprotective roles, CryAB suppressed the hypertrophy induced by short-term PO in the heart (Kumarapeli et al. 2008). Consistent with this, we also found higher levels of expression of CryAB, HSPB6, and HSPB1 that might alter QPR in congestive failing hearts.

A significant number of reports also support the notion that mitochondrial dysfunction plays a critical role in the pathogenesis of HF (O'Rourke et al. 2001; Russel et al. 2005; Ventura-Clapier^2009^; Hollander et al. 2011). Mitochondrial oxidative stress is also important in TAC-induced HF (Bugger et al. 2010; Dai et al. 2012). Here, in both genotypes, TAC-induced congestive failing hearts exhibited an aggravated downregulation of distinct mitochondrial protein sets which might cause an imbalance in energy supply and an increase in ROS production, affecting mitochondrial function through peroxidation. For instance, the overexpression of PRDX3, a mitochondrial antioxidant, prevents LV remodeling and failure after myocardial infarction in mice (Matsushima et al. 2006). Needless to say, the decrease in PRDX3 and heat shock protein (CH60) observed here might be detrimental for mitochondria, suggesting that congestive failing hearts may be more sensitive to oxidative stress. Although mitochondrial-targeted antioxidants have proved protective in various animal models of disease, they still await positive clinical trials (Griffiths 2012).

Our bioinformatic analysis highlighted a network in which huntingtin protein was found to be at the center of a cluster of mitochondrial proteins (Fig. 7A), but its precise function in heart disease remains to be elucidated. Here, we validate by Western blot analysis that TAC-induced cardiac HTT expression level was upregulated in HF. In addition, the IAP-derived interaction of the HTT with HSPB1 in heart was confirmed by coimmunoprecipitation experiments. However, we did not verify all interactomes with HTT. In order to better understand the involvement of HTT in the progression to HF, further functional experiments are required. When mutant huntingtin protein is expressed only in mouse cardiomyocytes (Pattison et al. 2008), the animals develop HF whereas knocking down huntingtin expression in mice (Zeitling et al. 1995) and in zebrafish (Lumsden et al. 2007) is embryonically lethal, resulting in defects in all three germ layers and in iron utilization, respectively. The notion that huntingtin protein may damage neurons by directly interfering with mitochondrial function in Huntington's disease (Cattaneo and Calabresi 2002), allows us to suggest that such a similar chain of events may occur in HF. Huntingtin protein appeared as a key node between mitochondrial dysfunction and cytoskeletal remodeling (TMG2). It is tempting to hypothesize that the increase in TGM2 and the dependence on the degree of the HTT polyglutamine expansion might contribute to more susceptibility to apopain cleavage leading to cytotoxic effects and likely to result in accelerated dysfunction in cellular energy metabolism. Speculatively, we suggest that huntingtin protein will become a new potential target for pathogenic mechanisms involved in HF progression.

However, mitochondria are also involved in intracellular Ca^2+^ handling. Mitochondrial Ca^2+^-transport is important in the generation of ROS and in the opening of the mitochondrial permeability transition pore, a factor potentially involved in HF (Griffiths 2012).

As a consequence of oxidative stress, mitochondrial alterations and functional impairment might lead to Ca^2+^ leak which, in turn, could directly interfere with the regulation of endoplasmic reticulum (ER) Ca^2+^-cycling. Thus, the ER might be overloaded with Ca^2+^ in congestive HF. A disturbance of Ca^2+^-homeostasis and overexpression of normal and/or incorrectly folded proteins interfere with ER function (Minamino and Kitakaze 2010). We also confirmed an increase in both ER stress proteins (ERP29, GRP78). Indeed, a marked increase in GRP78 involved in activation of the unfolded protein response (UPR) has been associated with the pathophysiology of human HF (Dally et al. 2009) and in mouse PO hearts (Okada et al. 2004). Another chaperone, HSP90, involved in UPR and regulation of apoptotic signaling through interaction with the proteins involved in the degradation process (Patterson and Cyr 2002), was also increased in failing hearts. These findings reflected an ER stress that might impair the UPR in H mice. Like the ER, the SR which is considered as a reservoir for Ca^2+^ release via the RyR2 channel during systolic contraction, then for Ca^2+^ capture via SERCA2a during relaxation, can also be stressed, either by altered SR Ca^2+^-homeostasis and/or by altered proteins which trigger cardiac dysfunction. A recent report associated ER stress with a reduced abundance of SERCA2a (Liu et al. 2011). We previously showed that altered Ca^2+^-handling proteins were associated with altered systolic and diastolic LV function in TAC mice (Prévilon et al. 2011). If the lumen of ER and SR is functionally well connected, the downregulation of SERCA2a might thus result in a reduced Ca^2+^ store in ER of congestive failing hearts. However, it remains to be demonstrated that ER and SR share a common Ca^2+^ store. Indeed, the concept that excessive levels of ROS/RNS (reactive nitrogen species) (Fassett et al. 2011; Tsutsui et al. 2011) and abnormal Ca^2+^-handling (Bers 2008) contribute to the development of contractile dysfunction in congestive HF is well accepted. ROS/RNS, normally produced in the heart, promote endogenous reversible RyR2-S-nitrosylation and S-glutathionylation (Donoso et al. 2011). Lastly, GSTO1 and ion chloride channel (CLIC1) bind to RyR channels (Donoso et al. 2011; Dulhunty et al. 2011). Both binding sites are on the clamp region of RyR2, a region that undergoes significant structural changes with channel opening and which bind another important regulator, FKBP12.6 (Donoso et al. 2011). Especially, the dissociation of FKBP12.6 from RyR2 caused Ca^2+^ leak via RyR2 and has been implicated in phenotypic changes in HF (Marks et al., 2002; Huang et al. 2006). We found that GSTO1 and CLIC1 protein levels were upregulated in failing hearts from mice of both genotypes. Thus, in such a context, these proteins might also modulate RyR2 activity in order to maintain low levels of Ca^2+^ leak during diastole, which might prevent arrhythmia and sudden cardiac death. Galfré et al.(2012) also highlighted some controversy over the functional effects of FKBP12.6 as the only channel stabilizer of RyR2. Furthermore, among the multiple molecular partners carefully orchestrating Ca^2+^-homeostasis, we also found an enhanced ANXA5 protein expression level in H mice, in agreement with its increased expression in failing human hearts, and its relation to systolic dysfunction in hypertensive patients (Camors et al. 2005, 2006; Ravassa et al. 2007). It also acted as a regulatory factor of Ca^2+^-handling proteins when it formed a complex with the Na^+^/Ca^2+^ -exchanger. Therefore, it is tempting to speculate that ANXA5 might contribute to impaired systolic function in mouse congestive HF.

## Conclusion

This study demonstrates distinct changes in the pathological LVH protein profile in response to TAC. The overexpression of FKBP12.6 is not sufficient to confer a sustained protection against chronic systolic overload. Compelling evidence supports a relationship between SR/ER and mitochondria which likely involve several Ca^2+^ and ROS-sensitive factors in the pathophysiology of end-stage HF. We speculate that huntingtin protein will become a new potential target for pathogenic mechanisms involved in HF progression. In TAC mice, huntingtin protein upregulation was related with a remarkable downregulation of proteins involved in energy metabolism. Although our understanding of the failing heart proteome has progressed in recent years, many important issues are still unresolved. As a consequence of PO, multiple cross-talks between organs can occur differently; therefore an integrative pathophysiology at the body level remains also to be explored. Nevertheless, in order to identify the pathways and to prove their mediating role, a large number of further cell biology and animal experiments are required that will provide us with new targets for drug discovery and therapeutic intervention.
